# Comparing the use of direct observation, standardized patients and exit interviews in low- and middle-income countries: a systematic review of methods of assessing quality of primary care

**DOI:** 10.1093/heapol/czaa152

**Published:** 2020-12-12

**Authors:** Navneet Aujla, Yen-Fu Chen, Yasara Samarakoon, Anna Wilson, Natalia Grolmusová, Abimbola Ayorinde, Timothy P Hofer, Frances Griffiths, Celia Brown, Paramjit Gill, Christian Mallen, Jo Sartori, Richard J Lilford

**Affiliations:** W-CAHRD, Warwick Medical School, University of Warwick, Coventry CV4 7AL, UK; W-CAHRD, Warwick Medical School, University of Warwick, Coventry CV4 7AL, UK; W-CAHRD, Warwick Medical School, University of Warwick, Coventry CV4 7AL, UK; W-CAHRD, Warwick Medical School, University of Warwick, Coventry CV4 7AL, UK; W-CAHRD, Warwick Medical School, University of Warwick, Coventry CV4 7AL, UK; W-CAHRD, Warwick Medical School, University of Warwick, Coventry CV4 7AL, UK; Department of Medicine, UM Institute for Health Policy and Innovation, Building 16 3rd Floor, North Campus Research Centre, University of Michigan Medical School, Ann Arbor, MI 48109-2800 USA; W-CAHRD, Warwick Medical School, University of Warwick, Coventry CV4 7AL, UK; W-CAHRD, Warwick Medical School, University of Warwick, Coventry CV4 7AL, UK; W-CAHRD, Warwick Medical School, University of Warwick, Coventry CV4 7AL, UK; Keele School of Medicine, David Wetherall Building, Keele University, Keele, ST5 5BG, UK; Institute of Applied Health Research, University of Birmingham, Edgbaston, Birmingham, B15 2TT, UK; Institute of Applied Health Research, University of Birmingham, Edgbaston, Birmingham, B15 2TT, UK

**Keywords:** Healthcare quality, technical competence, low-middle-income countries, healthcare quality assessment

## Abstract

Clinical records in primary healthcare settings in low- and middle-income countries (LMIC) are often lacking or of too poor quality to accurately assess what happens during the patient consultation. We examined the most common methods for assessing healthcare workers’ clinical behaviour: direct observation, standardized patients and patient/healthcare worker exit interview. The comparative feasibility, acceptability, reliability, validity and practicalities of using these methods in this setting are unclear. We systematically review and synthesize the evidence to compare and contrast the advantages and disadvantages of each method. We include studies in LMICs where methods have been directly compared and systematic and narrative reviews of each method. We searched several electronic databases and focused on real-life (not educational) primary healthcare encounters. The most recent update to the search for direct comparison studies was November 2019. We updated the search for systematic and narrative reviews on the standardized patient method in March 2020 and expanded it to all methods. Search strategies combined indexed terms and keywords. We searched reference lists of eligible articles and sourced additional references from relevant review articles. Titles and abstracts were independently screened by two reviewers and discrepancies resolved through discussion. Data were iteratively coded according to pre-defined categories and synthesized. We included 13 direct comparison studies and eight systematic and narrative reviews. We found that no method was clearly superior to the others—each has pros and cons and may assess different aspects of quality of care provision by healthcare workers. All methods require careful preparation, though the exact domain of quality assessed and ethics and selection and training of personnel are nuanced and the methods were subject to different biases. The differential strengths suggest that individual methods should be used strategically based on the research question or in combination for comprehensive global assessments of quality.

**KEY MESSAGES**

- Accurate measurement of healthcare workers’ clinical behaviour is crucial for improving clinical practice in primary care in low- and middle-income countries, where quality of care provision is reportedly poor and audit of clinical records is rarely possible.
- This paper is the first comparative overview of the most common methods and found that none are ‘gold standard’, contrary to existing suggestions.
- Each method has strengths and weaknesses and may assess different aspects of quality of care.
- Future selection and implementation of methods by policymakers, medical educators and researchers will rely more on feasibility and practicality and when used together can provide global quality assessment.

## Background

Improving healthcare quality is a major global public health challenge particularly in low- and middle-income countries (LMICs) (United Nations, 2015) and a recent report argues that quality of care has overtaken access to healthcare as the largest problem facing health systems in LMICs (Kruk *et al.*, 2018). **High**-quality healthcare is an essential pillar of Universal Health Coverage and target of the United Nations’ (UN) Sustainable Development Goal (SDG) 3 (United Nations, 2015). Most care is delivered in primary care and a large proportion of secondary care is based on referral from primary care. Poor quality of care provision by healthcare workers (doctors, pharmacists) in primary care in LMICs has been evidenced in many studies (Das and Sohnesen, 2007; Das *et al.*, 2008, 2012, 2015; Daniels *et al.*, 2017; Kwan *et al.*, 2018). Improving the quality of primary healthcare in LMICs is a current priority (World Health Organisation, 1978; Chabot, 1988; World Health Organisation, 2018, 2019).

It is difficult to assess the quality of primary care in an LMICs setting. In high-income countries (HICs), clinical records or databases are often used for this purpose but in LMICs, these data can be poor quality or incomplete, and depending on where patients consult, may be lacking entirely (Lilford *et al.*, 2007; Brown *et al.*, 2008; Luna *et al.*, 2013). Donabedian (1966) suggests that quality of care can be assessed in terms of structure, process and outcome, and described a causal chain linking structure to process and hence outcome. In this paper, we concentrate on process, which can be broken down into processes carried out at the system level, such as use of audit and feedback or improving staff morale, and clinical processes impacting directly on patients, such as questions asked to make a diagnosis or prescribe a treatment (Lilford *et al.*, 2010). We refer to the latter as the technical quality of care, corresponding with the definition provided by Donabedian (1988).

A number of methods have been used to assess the technical quality of healthcare. Miller (1990) argued that there are differences between what providers *know*, *know how* or *show how* to do in an examination setting and what they *actually do* in a real-life clinical encounter. The use of vignettes alone—written case descriptions—can only provide an assessment of the former, we will instead focus on three methods that assess the real-life delivery of care:

- Exit interviews/questionnaires: patients/carers/healthcare workers asked post-consultation about the provision of care in the consultation (Franco *et al.*, 2002; Schoen *et al.*, 2004);
- Direct observation: clinical practice is observed first-hand during consultations or via video- or audio-recording (Stojan *et al.*, 2016); and
- Standardized patients: individuals trained to act as patients and simulate a set of symptoms/problems to portray a particular clinical case (Peabody *et al.*, 2000).

These methods have been used extensively in medical education for training medical students and postgraduate and practising doctors in a variety of settings in HICs for decades (Beullens *et al.*, 1997; Overeem *et al.*, 2007; Rethans *et al.*, 2007; Hrisos *et al.*, 2009). There is now a growing evidence base of their application in LMICs (Watson *et al.*, 2006; Xu *et al.*, 2012; King *et al.*, 2019; Kwan *et al.*, 2019). While many of the references cited above are systematic reviews of one of these three methods, a systematic examination of the relative merits and drawbacks between these methods in LMIC settings is lacking.

In this paper, we review studies that have directly compared two (or more) of these methods ‘head to head’ and synthesize existing systematic and narrative review evidence on each method. We present a comparative overview of the feasibility, acceptability, validity, reliability, ethics, resources and costs involved in using these methods in the LMIC primary care setting. Our goal is to compare and contrast the pros and cons of using these methods to provide a resource to guide the future use of these methods in this context.

## Methods

We carried out two systematic reviews: the first review focuses on primary studies carried out in LMICs that compare one or more of direct observation, standardized patients and exit interviews head to head (hereafter termed *Direct Comparison Studies*). The second review supplements these data in an overview of the existing systematic and narrative review evidence on each of the different methods (hereafter termed *Overview of Reviews*). The reviews were conducted in accordance with best practice guidelines from the Cochrane Collaboration (Higgins and Green, 2011), and have been reported using the guidance published in the PRISMA statement (Moher *et al.*, 2009).

### Protocol and registration

The systematic review protocol is registered on the Prospero register (CRD42018088226).

### Search strategy

We performed searches using the following electronic databases: MEDLINE (from 1946), PsycINFO (from 1967), EMBASE (from 1980), CINAHL (from 1981), ASSIA (from 1987) and the Cochrane Library (from 1995). We first carried out searches to collate the *Direct Comparison Studies* in November 2018 and updated these searches in November 2019. We carried out the *Overview of Reviews* search in February 2018 and initially focused on the standardized patient method. The search was updated and expanded to all methods of interest in March 2020.

The search strategies used both indexed terms and keywords relating to important concepts of the review, including general terms related to healthcare quality and specific terms related to each of the three methods of assessing care quality. We tailored searches to the individual requirements of each database and applied an LMIC filter from the Cochrane Effective Practice and Organisation of Care (EPOC) review group (https://epoc.cochrane.org/lmic-filters) for the *Direct Comparison Studies’* search. We used truncations, wildcards and proximity operators where appropriate in all searches. The searches for the *Overview of Reviews* were restricted to review articles. Detailed search strategies can be found in the Supplementary Appendix.

### Eligibility criteria and study selection

Titles and abstracts retrieved were assessed independently by two reviewers against the inclusion criteria. The inclusion criteria for the *Direct Comparison Studies* review were as follows:

- Primarily concerns the technical quality of healthcare;
- Involves at least one comparison between direct observation, standardized patients or exit interview;
- Method has been applied to a primary or outpatient care encounter in a real life rather than educational setting; and
- Reports on a primary research study carried out in an LMICs setting.

The inclusion criteria for the *Overview of Reviews* were:

- Primarily concerns the technical quality of healthcare;
- Involves direct observation, standardized patients or exit interview;
- Method has been applied to a primary or outpatient care encounter in a real life rather than educational setting;
- Systematic or narrative review; and
- Provides empirical evidence on feasibility, acceptability, validity, reliability, ethics, resources and/or costs of the method(s).

While we focus on studies in which the quality-of-care assessment methods were directly compared in LMICs settings, we intentionally include review articles that have summarized literature related to application of these methods in both LMICs and HICs in order to cover a wider evidence base, as many features, strengths and weaknesses of each method hold true across different settings. An English language restriction was applied during study selection. No other restrictions were applied. Reference lists of included papers and other published reviews were hand searched to identify additional references. Duplicate references were removed. Discrepancies between reviewers’ decisions were resolved through discussion, with access to full-text papers available where necessary.

### Data extraction and synthesis

Data from studies confirmed to be eligible following the study selection process described above were extracted and coded according to a thematic framework covering several categories which were established a priori and refined during the data collection process. We extracted data separately for the *Direct Comparison Studies* and the *Overview of Reviews* though used the same thematic framework. The final categories were: country, location and setting; study design and sampling of patient, healthcare provider and healthcare facility; recruitment method and sample sizes (i.e. number of patients, healthcare providers, facilities and clinical encounters); sample characteristics; medical conditions or services involved; method of assessing care quality (including data collection tools); and training of study personnel. We also recorded information on feasibility, acceptability from the patient and provider perspective, practicality (including, ethical considerations, costs and resources required); inter and intra-rater reliability; content validity; criterion validity (measures of agreement between different methods or measures of accuracy of one method judged against another method/reference standard); and detection rate for the standardized patient method. Data extraction was undertaken by one reviewer and checked by a second reviewer, who together with a third reviewer derived the main themes for each of the data categories, which we used to construct summary tables and inform the narratives in this paper.

In order to establish the level of agreement between methods, different methods should ideally be deployed for the same consultation and findings from different methods can be compared with all other things being held equal. However, we noticed that in some of the included studies, measures of quality of care were taken using different methods during different consultations, and then the findings from the methods (based on different consultations) were compared using healthcare worker as the unit of analysis. In these cases, measurements obtained by different methods could be influenced by differences in the nature of individual consultations (e.g. patient’s presenting symptoms, health literacy, expectation, etc.). Consequently, it is difficult to attribute any observed disagreements to either the characteristics of the methods or the characteristics of individual consultations. We therefore made a clear distinction between these two types of studies, with more emphasis placed on the former which we term *within-consultation comparisons* (with individual consultation as the unit of analysis). Where a method did not share features with the other methods examined such as ethics of standardized patients or intrusiveness of an observer, the differences between the methods were highlighted in our descriptive analysis but were not possible to compare head to head.

## Results

### Study selection

**Figure 1:**
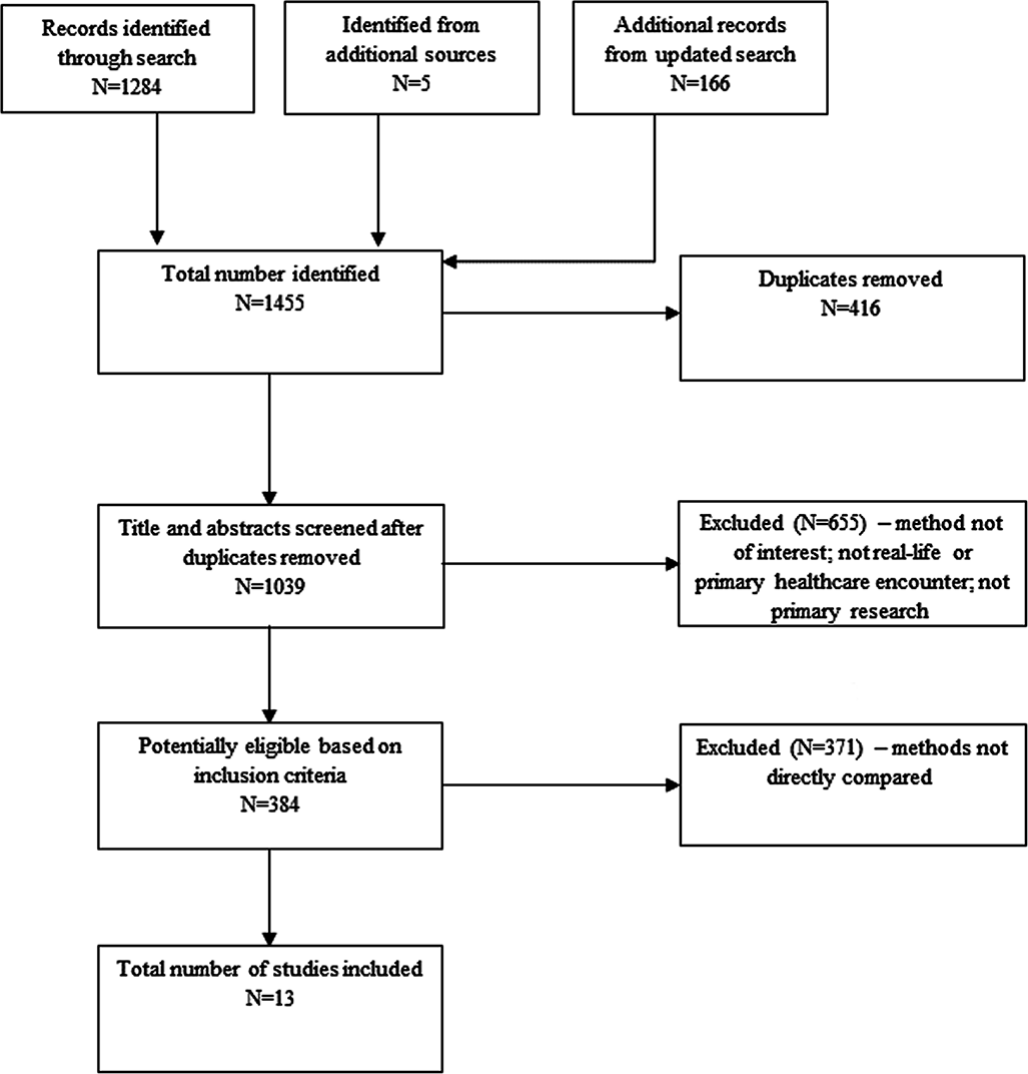
PRISMA diagram of the process of study selection for the Direct Comparison Studies.

**Figure 2:**
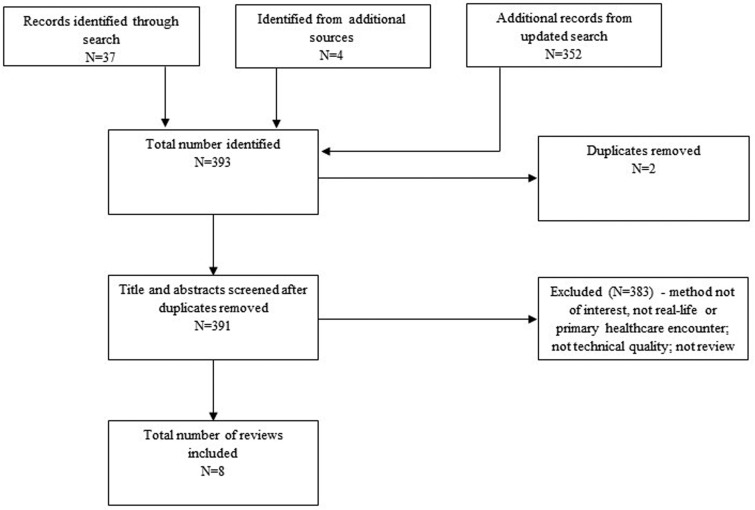
PRISMA diagram of the process of study selection for the Overview of Reviews.

The study selection process for each review is illustrated in Figures 1 and 2. Of 1455 records identified in the *Direct Comparison Studies* review, we removed 416 duplicates and screened 1039 titles and abstracts for eligibility. Thirteen studies met the pre-defined criteria for inclusion and are summarized in Table 1. Of 393 records identified in the *Overview of Reviews*, we screened 391 for eligibility after removing two duplicates. Eight reviews met the pre-defined criteria for inclusion and are summarized in Table 2.

**Table 1:** Characteristics of direct comparison studies included in the review

					Methods compared (see footnote for key)						
Author (year), country	Services	Type and number of institutions	Type and number of healthcare providers	Number of clinical encounters	DO + RE	DO	SP	EX (pt)	EX (pr)	RR^a^	VIN^a^
Assaf (2018), Haiti, Malawi and Senegal	Antenatal care	Formal-sector health facilities: Haiti (*n* = 905), Malawi (*n* = 977), Senegal (*n* = 363)		4899 Haiti: 1620, Malawi: 2068, Senegal: 1211		●		●			
Bessinger and Bertrand (2001), Ecuador, Uganda and Zimbabwe	Family planning services	Family planning facilities: Ecuador (*n* = 43), Uganda (*n* = 72), Zimbabwe (*n* = 39)	Consultations for family planning services (*n* = −)	1858 Ecuador: 583, Uganda: 539, Zimbabwe: 736		●		●			
Cardemil *et al.* (2012), Malawi	Management of sick children	Community health workers’ own facility (*n* = 131)	Community health workers (*n* = 131)	Child with cough and fast breathing (suspected pneumonia): 262, fever (suspected malaria): 262, diarrhoea: 393	●	●				●	●
Franco *et al.* (1997), Malawi	Sexually transmitted diseases case management	Randomly selected outpatient departments at three types of public and private health facilities (*n* = 39)	Providers directly observed and interviewed (*n* = 49); same providers visited by simulated patients (*n* = 20)	137		●	●		●		
Franco *et al.* (2002), Malawi	Outpatient paediatric care	Health centres (*n* = 12) and rural hospitals (*n* = 2)	‘Providers’ (*n* = 30)	Directly observed : 436, with 222 cough, 221 fever, 128 diarrhoea 426 Patient exit interviews		●		●	●	●	
Hermida *et al.* (1999), Guatemala	Acute ambulatory care and family planning	Ambulatory health centres (*n* = 3)	Health workers (physicians in most cases; also nurses and nursing auxiliary)	Respiratory infections: 74 and acute diarrhoea: 58 in children, and family planning counselling: 67		●●^b^		●		●	
Leonard and Masatu (2006), Tanzania	Common outpatient illnesses (fever, cough, diarrhoea)	Outpatient clinics (*n* = 45, but sub-sample of 11)	‘Clinicians’ (*n* = 107 and *n* = 12 in the sub-sample)	1100 DO 136 DO and EX (pt)^c^ 211 EX (pt)		●^c^		●^c^			
McCarthy *et al.* (2018), Kenya and Swaziland	Post-natal care	Eastern Kenya: hospitals (*n* = 4) and health centres (*n* = 8) Swaziland: public health units/MCH-FP (*n* = 8)	‘Healthcare providers’ (*n* = −)	Kenya: 545 Swaziland: 319		●		●			
Miller *et al.* (2015), Ethiopia	Management of sick children (mostly diarrhoea, malnutrition but some malaria, pneumonia, measles and severe illness)	Rural health posts (*n* = 103)	Health extension workers (*n* = 137)	257 directly observed and re-examined plus 544 from patient registers	●	●				●	
Onishi *et al.* (2011), Afghanistan	Outpatient consultations for children under 5 years	Randomly selected health facilities (>620 annually between 2005–07)	Doctors (44%), nurses (27.6%), midwives (13.9%), others (16.5%)	8659		●		●			
Pulford *et al.* (2014), Papua New Guinea	Malaria case management	110 health facilities	Nurses and community health workers (*n* = −)	1654 patients		●		●			
Rowe (2012), Benin	Management of child illnesses	55 public (n=47) and private (n=8) health facilities	89 health workers, trained and not trained in IMCI	54 SP visits and 185 DO	●		●				
Tumlinson *et al.* (2014), Kenya	Family planning service	19 public and private medium- to high-volume health care facilities (*n* = 108 family planning service providers)	SP and provider exit interview (*n* = 49); SP and DO (*n*-44); SP and patient exit interview (*n* = 31)	Not reported		●	●	●	●		

Key: Methods for assessing quality of care: DO + RE, direct observation with re-examination; DO, direct observation (without re-examination); SP, standardized patient; EX (pt), exit interview with patient/carer; EX (pr), exit interview with care provider; RR, record review; VIN, vignette; IMCI, Integrated Management of Childhood Illnesses.

^a^ RR and VIN were used in some of the studies but were not examined in detail in the current review, which is focused on DO, SP and EX.

^b^ Direct observation by experts vs direct observation by trained observers.

^c^ Direct observation and exit interview of patients were directly compared in consultations with clinicians from the sub-sample. Consultations with additional clinicians were used as part of the examination of the Hawthorne effect.

**Table 2:** Characteristics of systematic and narrative reviews included in the review

					Method (see footnote for key)								
Author (year), country	Services and type of healthcare provider	Number and nature of included papers (studies)	Type of institutions	Focus of review	DO + RE	DO	SP	EX (pt)	EX (pr)	RR^a^	VIN^a^	PR^b^	PRT^b^
Beullens *et al.* (1997), nine countries (mainly USA, Canada, the Netherlands)	General practice; type of provider not reported but likely GPs	31 (25 research reports and 6 theoretical discussions)	Primary care, −	Advantages, disadvantages, reliability and validity of SP method			●						
Hrisos *et al.* (2009), USA (*n* = 10), the Netherlands (*n* = 2), UK (*n* = 1), Australia (*n* = 1), Canada (*n* = 1)	Outpatient or paediatric care; mostly primary care physicians, but also nurses, community pharmacists and paediatricians	15 (reporting findings of different analyses of 11 studies providing data on comparative accuracy between methods)	Community or general internal primary care outpatient clinics, university medical centre, and general practices	Comparison of accuracy of direct measures (SP, DO) with proxy measures (EX, RR, VIN) of clinical behaviour		●^c^	●	●	●	●	●		
Overeem *et al.* (2007), USA, Canada, the Netherlands, UK	Routine practice; family doctors, hospital-based specialists and/or registrars	64 (report 58 observational studies of various design)	Both general practice and hospital settings.	Studies aimed at assessing individual doctors’ performance in routine practice; excluding studies using patient-based assessment tools only		●^d^	●			●		●	●
Rethans *et al.* (2007), USA, Canada, the Netherlands, Norway, UK	Mostly routine care; family doctors, general practitioners/primary care doctors, postgraduate specialists and residents	40 (report three experimental design but the rest descriptive)	Mostly primary care (three studies in secondary care)	Overview of studies that have used reliable and valid incognito standardized patient methodology to assess actual practice of doctors			●						
Watson *et al.* (2006), Europe (*n* = 13), Asia (*n* = 9), South and Central America (*n* = 8), North America (*n* = 7), Africa (*n* = 4); Australasia (*n* = 3), Middle East (*n* = 1), Bangladesh/Sri Lanka/Yemen (*n* = 1)	Supply of prescribed and over-the-counter medicines and provision of advice and counselling; pharmacists, pharmacy staff, drug sellers	46 (12 trials, 30 cross-sectional, 2 time-series and 2 ‘other’ designs)	Community pharmacy and drug stores	Definitive review of standardized patient methodology for use in pharmacy practice and identify key features for consideration in future studies			●						
Xu *et al*. (2012), Australia (*n* = 8), UK (*n* = 7), USA (*n* = 3), Canada (*n* = 2), Germany (*n* = 2), New Zealand (*n* = 1), Belgium (*n* = 1), Malaysia (*n* = 1), Kenya (*n* = 1), Peru (*n* = 1), Switzerland (*n* = 1), Norway (*n* = 1), Finland (*n* = 1)	Provision of non-prescription medicines; pharmacists and their staff	31, mostly cross-sectional, trials and pre–post	Community pharmacy	Explore use of standardized patient methodology in community pharmacy: purpose, types of scenarios employed, delivery of performance feedback, and acceptability to pharmacists			●						
King *et al.* (2019), LMICs	Routine care	Narrative review, but refer to 56 studies undertaken in a range of conditions (sexual and reproductive health, infectious diseases, non-communicable diseases, psychological and child infectious diseases)	Examples provided are from primary care field research	Discuss key steps in designing and undertaking standardized patient studies in health facilities in LMICs and methodological and ethical challenges of using this approach in this setting			●						
Kwan *et al.* (2019), LMICs with specific examples from China, India, Kenya and South Africa	Routine care	Narrative review, but refer to 10 studies undertaken in asthma, childhood diarrhoea, tuberculosis, unstable angina	Examples provided are from primary care field research	Conceptual framework for undertaking quality of care research and examining variation in quality in LMICs, drawing on real-life research examples. Also provide a manual and toolkit for the method			●						

Key: Methods for assessing quality of care: DO + RE, direct observation with re-examination; DO, direct observation (without re-examination); SP, standardized patient; EX (pt), exit interview with patient/carer; EX (pr), exit interview with care provider; RR, record review; VIN, vignette; PR, peer assessment; PRT, portfolio or appraisal.

^a^ RR and VIN were used in some of the studies but were not examined in detail in the current review, which is focused on DO, SP and EX.

^b^ Peer assessment (PR) and portfolio or appraisal (PRT) used by one study but were not examined in detail in the current review, which is focused on DO, SP and EX.

^c^ Includes direct in-person and audio or video-observation.

^d^ Includes direct in-person and video-observation only.

### Characteristics of included studies and reviews

#### Direct comparison studies

The characteristics of studies that directly compared quality of care assessment methods are summarized in Table 1. The studies were conducted in many LMICs worldwide though 10 out of the 13 took place in Sub-Saharan Africa. The healthcare settings included four family planning, antenatal and post-natal care; three community care; and five outpatient care services. One study covered both family planning and outpatient care (Hermida *et al.*, 1999). Outpatient services provided care for fever, malaria, diarrhoea, malnutrition, cough and pneumonia. Healthcare providers included doctors, nurses and nursing auxiliary, midwives and community health workers. Six studies were carried out with adult patients and five with children. Two studies included both adult and child patients (Leonard and Masatu, 2006; Pulford *et al.*, 2014). Included studies covered around 3600 healthcare settings and just over 21 000 clinical encounters overall. The number of healthcare providers included was not reported in 5 out of 13 papers, though the remainder included 651 healthcare providers.

#### Overview of reviews

We included six systematic and two narrative reviews, which are summarized in Table 2. Studies included in four of the six systematic reviews took place in HICs (USA, Canada, the Netherlands, Australia, Norway and UK). Studies included in the remaining two systematic reviews covered both HICs and LMICs and overall most were conducted in Asia or Central and South America. The six systematic reviews included 227 papers overall and these covered routine care mostly in general practice or pharmacy settings with family doctors/general practitioners, pharmacists or pharmacy staff and drug sellers. All six systematic reviews examined the use of the standardized patient method and three of these also examined direct observation and patient and provider exit interviews. Both narrative reviews examined the use of the standardized patient method in the LMICs context. They provide very detailed descriptions of issues and recommendations to be considered for adopting this method, drawing from extensive empirical evidence. Of the eight systematic and narrative reviews, quantitative comparisons between different methods were examined in one review (Hrisos *et al.*, 2009) and we consolidated these with the direct comparison studies included in our review.

We first present data from our analysis of the *Direct Comparison Studies*: these data summarize the quantitative comparisons between the different care quality assessment methods based on quantitative measures of agreement between the methods.

### Types of head-to-head methodological comparisons made

The most common comparison was between the direct observation and patient or healthcare worker exit interview methods (*n* = 8 studies). Two studies compared all three methods head to head (Franco *et al.*, 1997; Tumlinson *et al.*, 2014). A further two studies compared different types of direct observation: Miller *et al.* (2015) compared direct observation with repeat examination by a third party against direct observation alone and Cardemil *et al.* (2012) compared direct observation with repeat examination by expert examiners vs trained observers.

### Assessment tools/instruments employed in directly compared studies

A typical primary healthcare consultation can be broken down into the following processes: history taking, physical examination, diagnosis, treatment/management, advice/counselling and preventive measures (Byrne and Long, 1976). Each method can assess each of these parts of the clinical encounter and included studies typically employed checklists to facilitate these assessments. The checklists captured the required or desirable actions one would expect a healthcare worker to perform during a clinical encounter (such as asking history questions, checking a symptom, ordering a test and prescribing a medication) for a given symptom or condition. Most of the criteria had been selected in accordance with accepted local and international clinical standards. Most studies created their own scoring algorithms to score checklist criteria.

### Quantitative comparisons between different methods compared head to head

Here we examine quantitative measurements of agreement between the different methods described above. Comparisons between different methods can be viewed from two perspectives. The first perspective is to assume that one method is more accurate than the other method(s), and thus the former is used as a reference standard against which the ‘performance’ of the other methods is judged. The second perspective is to assume that different methods are broadly similar in terms of their validity, and therefore agreement between methods is measured to inform whether one method can be used in place of the other methods. Studies included in this review adopt either or both of these perspectives and these comparisons are summarized in Table 3.

**Table 3:** Reported levels of agreement between different methods for assessing quality of care in the included studies, sorted by comparisons

	Agreement (%)	Kappa	Sensitivity (%)^a^	Specificity (%)^b^	PPV (%)^c^	NPV (%)^d^	Area under ROC curve	LR+^e^			LR−^f^	Within-consultation comparison^g^
Direct observation vs reference standard (expert observation/re-examination)												
Cardemil *et al.* (2012)	43–97	0.15–0.92	32–93	42–99								
Hermida *et al.* (1999)			20–100^h^	39–98^h^								●
Miller *et al.* (2015)			34–100	30–100			0.54–0.90					●
Direct observation vs standardized patient												
Franco *et al.* (1997)	43–84	−0.18–0.47										
Tumlinson *et al.* (2014)	37–95		55–100	0–95	0–98	18–100		0.8–1.1			0.6–1.5	
Exit interview (patient/carer) vs direct observation												
Assaf (2018)	52–60	0.05–0.20	33–61^i^	54–72^i^								
Bessinger and Bertrand (2001)	55–99	0.08–0.99										●
Franco *et al.* (2002)	43–97	−0.28–0.91										●
Hermida *et al.* (1999)			40–97^h^	57–96^h^								●
Leonard and Masatu (2006)	57–77	0.17–0.28^j^	55–100^j^	0–72^i^	56–77^i^	53–76^i^			1.0–2.3^i^	0.5–0.8^i^		●
McCarthy *et al.* (2018)			49–97	33–91								●
Onishi *et al.* (2011)			33–83	63–90			0.61–0.77					●
Pulford *et al.* (2014)			36–98	54–99								●
Exit interview (patient/carer) vs standardized patient												
Tumlinson *et al.* (2014)	23–93		32–100	0–67	0–96	0–100		0.9–1.1			0.5–1.4	
Exit interview (provider) vs direct observation												
Franco *et al.* (1997)	31–96	−0.08–0.56										
Franco *et al.* (2002)	62–82	0.22–0.60										
Exit interview (provider) vs standardized patient												
Tumlinson *et al.* (2014)	45–94		50–98	6–83	8–100	5–96		0.6–1.0			0.9–4.0	

^a^ Sensitivity = (task performed and correctly recorded by the assessment method)/(all tasks performed based on reference standard).

^b^ Specificity = (task not performed and correctly identified as such by the assessment method)/(all tasks not performed based on reference standard).

^c^ PPV (positive predictive value) = (task identified as performed by both the assessment method and reference standard)/(all tasked identified as performed by the assessment method).

^d^ NPV (negative predictive value) = (task identified as not performed by both the assessment method and reference standard)/(all tasked identified as not performed by the assessment method).

^e^ LR+ (positive likelihood ratio) = sensitivity/(1—specificity).

^f^ LR− (negative likelihood ratio) = specificity/(1—Sensitivity).

^g^ Refers to comparisons made between methods based on the same clinical encounter (with the same provider-patient dyad) where quality of care is assessed using the same indicators.

^h^ Sensitivity and specificity were defined in an opposite way in this study (e.g. sensitivity was defined as performance failures detected by the assessment compared with reference standard) compared with the definitions adopted in this review as described in the footnotes above; figures presented in this table have been inverted on this basis to reflect the standard definitions adopted in this review.

^i^ Calculated based on data reported in the original paper.

^j^ Coefficient of correlation.

−, not reported.

As shown in the table, many studies reported measures of ‘accuracy’ of one method against a reference standard such as sensitivity, specificity, positive predictive value (PPV) and negative predictive value (NPV), receiver operating characteristic (ROC) curve and positive likelihood ratio (LR+) and negative likelihood ratios (LR−). Most of the studies also reported measures of agreement between methods such as percentage agreement or kappa statistics. Irrespective of the methods compared and measures reported, a common finding is that the levels of agreement between methods vary widely depending on the nature of the quality item (e.g. whether it relates to history taking, physical examination, diagnosis or giving advices) and the specific context (e.g. disease/service area, availability of medicines and diagnostic tests, patient’s condition, presentation, needs and health literacy). For example, the reported agreements typically ranged from between 30–60% at the lower end and over 90% at the upper end for different quality items within individual studies (Table 3). Agreement between different methods could also be influenced by methodological issues, such as the wording of survey questions and level of ‘probing’ when conducting the interview (Franco *et al.*, 1997). For example, Franco *et al.* (1997) reported different rates for performing required tasks from exit interviews with healthcare workers when only spontaneous answers were counted compared with inclusion of answers both offered spontaneously and after probing. The latter often resulted in higher rates of reported acts (e.g. for the item ‘advised (the patient) to finish treatment’ (for sexually transmitted diseases): 33% using spontaneous answers, 100% using both spontaneous and probed answers, vs 65% recorded in direct observation). However, the effect of probing and discrepancies between exit interviews and direct observations also appear to be item-specific and were not uniformly observed for all items.

We present below findings from pairwise comparisons between the methods and highlight pertinent methodological issues. In points (1) and (2) below, we first describe attempts to validate direct observation by comparing this approach with a reference standard perceived to be superior (in at least some aspects, such as a more accurate diagnosis through re-examination of the same patient by a more experienced/better-qualified person, or removing potential Hawthorne effect by using standardized patients). This is followed by comparison of patient/carer/healthcare worker exit interviews with these reference standards [points (3) and (4)].

#### (1) Validation of direct observation with re-examination and/or more experienced observers

Three studies provided evidence to attempt to validate direct observation by using an ‘improved’ version of this approach (see Table 3). Two of these three studies (Cardemil *et al.*, 2012; Miller *et al.*, 2015) presented what we term *within-consultation comparisons*, i.e. comparisons based on the same clinical encounter (with the same healthcare worker–patient dyad) where quality of care is assessed using the same indicators. These two studies compared direct observation without re-examination against direct observation with re-examination and the remaining study compared direct observation made by neophyte physician observers with observation made by experienced expert as the reference standard (Hermida *et al.*, 1999). Detailed findings are presented in Supplementary Appendix Table SA1.

The agreement between direct observation and reference standards (as noted in Table 3) was reported in one study and ranged from 43% to 97%, with kappa statistics spanning from −0.15 to 0.92. Judged against the reference standards, and focusing only on studies reporting *within-consultation comparisons*, direct observation demonstrated a sensitivity between 20% and 100% and a specificity between 30% and 100%, with the area under the ROC curve ranging from 0.54 to 0.90 were reported. Direct observation showed good agreement overall with reference standards, but a pattern consistent across studies was that its performance against reference standards tended to be much lower with respect to recognition and management of severe acute illness (Supplementary Appendix Table SA1).

#### (2) Direct observation vs standardized patients

Two studies, Franco *et al.* (1997) and Tumlinson *et al.* (2014) provided evidence for this comparison. Levels of agreement between standardized patients and direct observation were generally high in the two studies, although they ranged from 37% to 95% for individual items. Kappa statistics were reported only in Franco *et al.* (1997) and indicated moderate to poor agreement (range −0.18 to 0.50). Tumlinson *et al.* (2014) reported sensitivity (55–100%) and specificity (0–95%) along with PPV (0–98%), NPV (18–100%), LR+ (0.8–1.1) and LR− (0.6–1.5) (see Table 3).

Overall the prevalence of appropriate/correct responses for quality items reported by standardized patients tended to be similar or lower than that recorded through direct observation (see Supplementary Appendix Table SA1). However, the interpretation of findings from the studies requires great caution as in both studies the consultations assessed by standardized patients were not the same consultations being directly observed (i.e. the unit of analysis was providers rather than consultations), and therefore the observed discrepancies could be attributed to features of the consultations rather than the methods of assessment. One further study (Rowe *et al.*, 2012) compared the two methods using data aggregated across consultations. The analyses did not quantify agreement between the methods and were mainly undertaken to estimate the magnitude of Hawthorne effect (described later).

#### (3) Patient/carer exit interview vs direct observation or standardized patient

Nine out of 12 included studies assessed exit interview of service users (Hermida *et al.*, 1999; Bessinger and Bertrand, 2001; Franco *et al.*, 2002; Leonard and Masatu, 2006; Onishi *et al.*, 2011; Pulford *et al.*, 2014; Tumlinson *et al.*, 2014; Assaf, 2018; McCarthy *et al.*, 2018). Seven of these studies reported *within-consultation comparisons*. The reference standard was direct observation in eight studies and standardized patients in Tumlinson *et al.* (2014). Findings of these studies are presented in Supplementary Appendix Table SA2.

Levels of agreement ranged from 23% to 99% and kappa ranged from −0.28 to 0.99. A wide range of sensitivity (33–100%), specificity (0–99%), PPV (0–96%), NPV (0–100%) and area under the ROC curve (0.61–0.77) was reported. One study (Onishi *et al.*, 2011) examined whether the performance of exit interviews varied by types of healthcare worker (doctors, nurses or midwives) but did not find any major differences. The study by McCarthy *et al.* (2018) examined the potential effect of women’s sociodemographic characteristics on the performance of exit interviews but also did not find an association.

We found that patients tend to remember some elements of the consultation better than others: they are more likely to remember things that are easily discernible from the encounter, such as being asked about a particular bothersome symptom (e.g. Have you noticed blood in your stool?). They are also more likely to recall actions that were done to them such as the healthcare worker asking for a stool sample or listening to their chest. Patients are much less likely to recall, or even recognize, the very technical or more abstract aspects of care, such as if the healthcare worker washed their hands or respected their confidentiality—these are elements more accurately picked up through observation of care (Bessinger and Bertrand, 2001). Patients might also remember the working diagnosis if shared with them by the healthcare worker and if they were given any counselling or specific advice, such as coming back immediately if breathing becomes difficult, or if the healthcare worker was rude or treated them disrespectfully (Onishi *et al.*, 2011). A further issue is that patient/carers’ responses might be influenced by the wording of the questions and their understanding of the procedures carried out/advice given to them by healthcare workers (Hermida *et al.*, 1999; McCarthy *et al.*, 2018), and could be confounded by knowledge that they already possessed or gained elsewhere outside the consultation (Bessinger and Bertrand, 2001).

#### (4) Healthcare worker exit interview vs direct observation or standardized patients

Three studies assessed healthcare worker interview: two compared with direct observation (Franco *et al.*, 1997, 2002) and one compared with standardized patients (Tumlinson *et al.*, 2014). Findings from these studies are presented in Supplementary Appendix Table SA3. Levels of agreement ranged from 31% to 96%, with reported kappas between −0.08 and 0.60. Only Tumlinson *et al.* (2014) reported sensitivity (50–98%), specificity (6–83%), PPV (8–100%), NPV (5–96%), LR+ (0.6–1.0) and LR− (0.9–4.0). In all three studies, the interview with healthcare workers might not have been directly linked to the specific consultations assessed by direct observation or standardized patients; these instead seemed to be carried out with healthcare workers after a set of observations took place. Without encouraging healthcare workers to reflect on what happened with a particular patient, a healthcare worker exit interview may rather be providing an assessment of knowledge of care rather than actual behaviour. Therefore, this approach may actually be equivalent to asking healthcare workers to complete a vignette about the clinical case.

### Descriptive comparisons between different methods

Now that we have compared methods based on measures of agreement, we turn to issues of feasibility, acceptability and practical considerations (ethics, resource use and cost) relevant to each method. We derived these data from all papers included across both elements of the review: the *Direct Comparison Studies* and the *Overview of Reviews*. Two themes emerged, which we name: method preparation and implementation, covering issues such as ethics, resources required and clinical case/selection of illnesses; and methodological issues covering validity/bias. We summarize the key issues for each of these themes in Table 4 and organize the issues according to whether they are advantages or disadvantages in the use of direct observation, standardized patients or patient/carer/healthcare worker exit interviews.

**Table 4:** Pros and cons of each quality of care assessment method to guide use in LMICs

	Direct observation	Standardized patients^a^	Patient/carer/provider exit interview
**Pros**	+ Flexible—used in-person or via audio or video-recording. + Easily transportable. + Used for both child and adult consultations. + Canvass a breadth of conditions. + Structured checklists with objective criteria can remove subjectivity when coding observations. + Reliable with either expert or trained neophyte observers.	+ Non-intrusive. + Assesses knowledge-do gaps. + Used extensively in pharmacies and primary care clinics in LMICs—comprehensive guidance and toolkit available to guide use in these settings. + Not affected by Hawthorne effect. + No social desirability bias. + Immediate post-visit completion of assessment checklists minimizes recall bias. + Low detection rate (<1% or 0– 0-5% in recent LMIC studies). + Low false positive rate—providers report real patients as being standardized patients—(1–6% in recent LMIC studies). + Reliable. + ‘In-principle’ consent can avoid ethical concerns. + Used in a breadth of both common and relatively rate outpatient symptoms/conditions possible to mimic. + Can be used with adults and for selected child conditions (e.g. malaria) with or without child present	+ Flexible—data collection via questionnaire or interview. *+* Not affected by Hawthorne effect. + Straightforward to implement. + Can be brief. + Minimal intrusion to health facility. + Supplements data collected using other methods. + Reliably provides information on quality of care from the patient/spouse/carer and provider perspectives. + Canvass a breadth of conditions. + Easily transportable. + Used for both child and adult consultations. + Used across full range of primary healthcare settings. + Patients good at recalling disrespectful treatment.
**Cons**	− Intrusive. − Requires significant buy-in from a range of stakeholders. − Limited information on acceptability amongst providers in LMICs. − In-person observation may be impractical to use in pharmacies. − Hawthorne effect, but people do habituate. − Resource intensive—requires multiple highly trained observers independent of the health facility. − Time-consuming to code observations. − Equipment failures possible. − Tends to assess only what the healthcare provider recommends, instead of effectiveness or appropriateness of care (but this may be possible with repeat examination). − Need to observe high numbers to ensure enough observations to compute quality scores for relatively rare symptom or conditions.	− Ethical debate around prior consent from healthcare providers. − Requires significant buy-in from a range of stakeholders. − Initial set-up resource (time, effort, finance) intensive. − Cases require careful selection—technically feasible, ethically acceptable, and suitable to local context. − Cannot be used for illnesses with physical signs (e.g. trauma, pregnancy) that cannot be mimicked. − Cannot be used where there are intimate, invasive or surgical procedures. − Requires carefully selected and highly trained standardized patients; particularly challenging if involving children. − Standardized patients must represent ‘typical’ patients for the specific context to ensure credibility and thus face and content validity. − ‘First-visit’ bias—leading to underestimated performance from one-time interactions; not suitable for assessing follow-up consultation of chronic conditions − Limited information on acceptability amongst providers in LMICs. − Visits sometimes made to the wrong premises and healthcare providers. − Samples of healthcare providers can be self-selected. − Visits capped at three per day to maintain reliability of post-visit checklist. − Need to purchase all drugs offered.	− Requires skilled field workers. − Self-reported—affected by recall bias, social desirability bias and courtesy bias. − Patients much less likely to recall or even recognize the very technical or more abstract aspects of care.

^a^ The longer list of pros and cons for the standardized patient approach does not display a preference for this approach over the others—the literature on use of this method in the context of this review is significantly more comprehensive and detailed than it is for the direct observation and exit interview approaches.

Details on the acceptability of the different methods were absent from the papers included in this review. Cost information was available in Rowe *et al.* (2012) and two of the reviews (Overeem *et al.*, 2007; Kwan *et al.*, 2019), but one of them (Overeem *et al.*, 2007) only focused on HICs. Rowe *et al.* (2012) reported similar costs per consultation of $73.67 and $70.19 for direct observation (with re-examination) and standardised patients, respectively. Kwan *et al.* (2019) offered detailed discussions on budgetary consideration for planning standardized patient methods in LMICs in their comprehensive Supplementary data. There are inevitable, substantial variations in cost estimates from previous studies depending on countries, settings and type of costs included (e.g. costs of out-of-country research/advisory teams), but they highlighted that the scale and complexity of individual projects have a major impact on estimated costs per patient–provider interaction, ranging from 60–150 US dollars in a project involving ~8000 interactions in India to 900–1000 US dollars in a smaller project involving around 400 interactions in South Africa. They further noted that the average cost per interaction decreased over time (in the above study in India) because the teams became more efficient with accumulation of experiences and the initially higher set-up costs were divided across more subsequent interactions.

### Method preparation and implementation

Preparatory work is required for all methods before implementation in clinical care. The standardized patient method benefits from the recently published comprehensive guidance and toolkit which describes how to implement this approach in practice in LMICs and covers all of the important considerations alongside exemplars and templates (King *et al.*, 2019; Kwan *et al.*, 2019). Comparable guidance is not available for the direct observation or exit interview methods.

Patient/carer/healthcare worker exit interviews are by far the most straightforward to implement in practice. The other approaches are complex to administer and resource intensive. Authors stressed the importance of carefully selecting and training field staff and is key for the standardized patient method in particular. There is some debate around the ethics approach of the standardized patient method, though the recommended approach is to seek ‘in-principle consent’ from healthcare workers before visits take place, i.e. permission to be visited by a standardized patient visit but not being told when it will happen. Rowe *et al.* (2012) highlighted some ethical and practical challenges when using the standardized patient method involving children, such as minimising potential harm and discomfort for them and dealing with relatively common occurrence of (real) acute illnesses for young children.

There is a clear trade-off between direct observation and exit interviews on the one hand and standardized patients on the other. Standardized patients have a distinct advantage over other methods because it is not necessary to wait for a case with one of the conditions of interest to present. For example, it may be necessary to screen large numbers of consultations to find one with a presenting feature such as loss of weight or a persistent cough while each standardized patient encounter would already include a condition of interest. But the price to pay is that suitable conditions are limited to those that can be represented by a standardized patient (i.e. non-emergency conditions that do not require invasive or intimate examinations or interventions, and that do not require sequential visits to or long term/continual care with a specific provider). Direct observation and exit interviews canvass a larger range of conditions, but these methods are likely to capture too small a number of relatively rare conditions to allow reliable assessment of quality of care.

### Methodological issues

The Hawthorne effect, which describes a change in behaviour as a result of being observed (Sommer, 1968), is a concern in direct observation. The suggestion when examined in five papers (Leonard and Masatu, 2006; Tumlinson *et al.*, 2014; Miller *et al.*, 2015; McCarthy *et al.*, 2018; Rowe *et al.*, 2012) is that it could lead to bias of the results in an upward direction (i.e. better performance than usual). Three studies attempted to quantify the Hawthorne effect (Leonard and Masatu, 2006; Miller *et al.*, 2015; Rowe *et al.*, 2012). Rowe *et al.* (2012) found a median difference of 16.4 percentage points higher (range 1.7% lower to 61.1% higher) for quality indicators assessed by direct observation compared with standardised patients. Miller *et al.* (2015) calculated the differences in point estimates of care quality indicators obtained from medical record review between children whose consultations were observed vs those not observed. The authors found only small differences between many of the quality indicators—most of which showed statistical non-significance—and concluded that the effect of being observed was negligible. However, the validity of the finding partly relies upon the accuracy of medical record review, which was found to have generally high sensitivity but low specificity in the same study. In contrast, Leonard and Masatu (2006) quantified the Hawthorne effect by comparing quality of care measures obtained through patient exit interviews that took place either before or after the research team arrived in clinic to observe care. The authors found an increase of 13 percentage points in quality of care (from baseline scores of just over 50%) at the beginning of direct observation (i.e. a Hawthorne effect). However, the initial improvements in quality gradually dissipated over time and returned to their baseline level after 10–15 observations. Therefore, one way to mitigate the Hawthorne effect might be to carry out multiple days of observations at healthcare facilities to help individuals habituate to being observed (McCarthy *et al.*, 2018).

Exit interviews and standardized patient approaches are not affected by the Hawthorne effect but as exit interview data are self-reported, patients/carers/healthcare workers’ responses can be affected by social desirability bias, courtesy bias and recall bias (Tumlinson *et al.*, 2014). This could again skew the data towards higher perceived quality of care. The unannounced design of standardized patient visits can reduce introducing the risks of the Hawthorne effect and/or social desirability bias.

While it might seem that healthcare workers could detect standardized patents, this has been shown to happen rarely (<1%) especially when the standardized patients blend in with the local patient demographic (Tumlinson *et al.*, 2014). That being said, some conditions have higher risk of discovery and Franco *et al.* (1997) stressed that standardized patients should be given clear instructions on when to abscond to maintain their cover. The authors used standardized patients in the context of sexually transmitted disease management and involved those who did not have the symptom (urethral discharge) for the diseases they were simulating. The danger here is that non-symptomatic standardized patients may be treated differently by healthcare workers compared with symptomatic patients. Standardized patients in this study absconded in 5 out of 20 consultations.

## Discussion

Improving the quality of primary healthcare provision is an important goal for many LMICs and a current WHO priority. While recent widespread efforts have been made to assess the quality of primary healthcare in LMICs, the measurement of consultation quality remains a challenge. We reviewed the most common methods for assessing healthcare workers’ clinical behaviour: direct observation, standardized patients and exit interviews. Our goal was to compare and contrast the pros and cons of each method and provide a resource to guide the selection of methods in this context in the future.

Direct observation and standardized patients are commonly considered to be ‘gold standard’ methods (Akachi and Kruk, 2017), though we did not find this to be the case. We found that no single method was superior to the other methods across the different contexts in our review. Each method may assess different aspects of quality of care provision and their differential strengths and weaknesses from a methodological and practical standpoint will most likely guide decisions on method selection.

We found that the accuracy and validity of an individual method for assessing quality of care are by no means fixed and may depend on the nature of the aspect/item of technical quality being assessed, but more crucially also rely on careful planning and implementation before and during the application of each method. The exact reasons behind the discrepancies in the accuracy and validity of these methods observed between different studies are not always clear and need to be investigated in further research. Until we have a better understanding, it is important that any chosen methods are cross-validated, possibly with at least another method in the setting in which they are to be deployed.

When comparing the accuracy of different methods in recalling what happened in consultations, *within-consultation comparisons* may provide the best evidence, as confounding arising from differences in the case mix and characteristics of patients between consultations is avoided. Nevertheless, there are inherent methodological challenges related to the difficulty in isolating the influence of one method (such as direct observation) from measurements made by another method. Potential interactions between methods of assessment and patient and healthcare workers’ behaviours (e.g. how they react during the consultation and what they recall after the consultation) therefore need to be taken into account when interpreting data from *within-consultation comparisons*. One particular concern is the Hawthorne effect that may be induced by direct observation. Findings from studies included in this review suggest that the Hawthorne effect associated with direct observation of patient consultation is likely to be small or moderate and tends to dissipate over time (Leonard and Masatu, 2006; Miller *et al.*, 2015; Rowe *et al.*, 2012). Approaches to minimizing a potential Hawthorne effect [such as having a longer period of observation until the care providers habituate to the presence of observers/recording mentioned earlier (McCarthy *et al.*, 2018)] may alleviate this problem, but these will inevitably increase resources required to undertake the observations.

The standardized patient method has several advantages including avoidance of the Hawthorne effect in direct observation and various biases associated with responses given by healthcare workers or patients. Standardized patients also overcome the difficulty in establishing a ‘correct diagnosis’ and hence the uncertainty in judging the appropriateness of subsequent decisions made by the healthcare worker for encounters with real patients. In addition, standardized patients provide a means to standardize patient characteristics during the consultations being assessed, thereby alleviating or abolishing the problem of confounding. Nevertheless, a limitation inherent to this method is the type of conditions and nature of the clinical problems to which it can be applied and the many practical challenges and costs described earlier.

The acceptability of each method from the perspective of relevant stakeholders (healthcare workers, patients, health facilities, etc.) was not considered in any of the papers included in our review but is crucial for ensuring ‘buy-in’ and the smooth-running of quality of care projects (Kwan *et al.*, 2019). Rethans *et al.* (2007) suggest that combining performance feedback with quality of care assessments may enhance perceived acceptability. Performance feedback was considered in only one of the systematic reviews we included in our review (Overeem *et al.*, 2007) and in only a third of their included studies. However, audit combined with the provision of feedback (so-called ‘audit and feedback’) is a well-established and effective strategy for improving healthcare workers clinical behaviour by making them aware of where the inconsistencies are in their clinical practice (Hysong, 2009; Ivers *et al.*, 2012; Rowe *et al.*, 2018). Although there may be an additional cost implication and need for a skilled facilitator if feedback is to be optimally effective.

### Review limitations

The findings are limited by the small number of available studies, which limits the generalizability of our quantitative comparisons. While we have focused on studies that directly compared at least two assessment methods, we are aware that there is a large body of literature in which individual methods were used singly to assess the quality of care in LMIC settings. While not providing direct comparative evidence, these may have described valuable practical lessons related to the planning and implementation of individual methods that may not have been captured in this review. This is partially compensated by our inclusion of two comprehensive narrative reviews on standardized patients, but we did not find similar reviews for direct observation and exit interviews.

The direct comparison studies we found were highly heterogeneous. Different measures were used to characterize the performance of different methods of assessment, which hinders the comparison of findings between studies. The studies were also diverse in the types of comparisons made and often did not compare the same clinical encounters or domains. We report *within-consultation comparisons* where available, i.e. comparisons made on the same clinical encounter where quality of care are assessed using the same indicators. Alternatively, comparisons may be made using different patients but assessing the same indicator of quality or different patients and different indicators of quality.

In this review, we have focused on comparing the fidelity of individual methods to capture what happens in individual consultations and the practical considerations in choosing between the methods. The ultimate goal in applying these methods is to ensure that quality of care can be reliably measured across the healthcare system, and that any deficiencies in the care can be detected and addressed. Evaluating the quality of care of individual consultations is therefore an essential building block but may not be sufficient on its own to achieve this goal. In order to produce reliable and comprehensive assessments, data on technical quality of care gathered from individual consultations will need to be supplemented by data describing the variation in average encounter quality at provider, facility and higher levels for any population targeted for measurement and potentially used alongside other data such as accessibility and patient experience.

Our review did not include studies investigating the use of vignettes because vignettes measure the healthcare workers’ knowledge rather than actual practice which is the focus of this review. Vignettes nevertheless remain a very important tool to establish knowledge-do gaps where problems with clinical practice are identified [see Mohanan *et al.* (2015)], and should therefore be considered alongside the methods considered in our review when planning a programme or research to evaluate and/or improve quality of care in the primary care setting (Peabody *et al.*, 2000; Das and Hammer, 2005; Leonard and Masatu, 2005).

## Conclusion

No single method was superior to the others for assessing the technical quality of healthcare in primary care in LMICs. At an individual patient level, there are little data to estimate consistency of measurement by the different methods, or to identify one as ‘gold standard’. Individual methods should be used strategically based on the research question and necessarily, the choice of method will rely more on the feasibility and practicality. It may also be worth considering the approaches as potentially complementary and where possible, include some or all of the methods to capture the full spectrum of quality of care.

## Supplementary data

Supplementary data are available at *Health Policy and Planning* online.

## Funding

This research was funded by the National Institute for Health Research (NIHR) Global Health Research Unit on Improving Health in Slums (16/136/87) using UK aid from the UK Government to support global health research. RJL and PG are supported by the National Institute for Health Research Collaboration for Leadership in Applied Health Research and Care West Midlands (NIHR CLAHRC WM), now recommissioned as NIHR Applied Research Collaboration West Midlands (NIHR200165).The views expressed in this publication are those of the author(s) and not necessarily those of the NIHR or the UK Department of Health and Social Care.

*Conflict of interest statement*. None declared.

*Ethical approval*. No ethical approval was required for this study.

## Supplementary Material

czaa152_Supp
