# Corrigendum to: comparing the use of direct observation, standardized patients and exit interviews in low- and middle-income countries: a systematic review of methods of assessing quality of primary care

**DOI:** 10.1093/heapol/czab046

**Published:** 2021-05-14

**Authors:** Navneet Aujla, Yen-Fu Chen, Yasara Samarakoon, Anna Wilson, Natalia Grolmusova, Abimbola Ayorinde, Timothy P Hofer, Frances Griffiths, Celia Brown, Paramjit Gill, Christian Mallen, J o Sartori, Richard J Lilford

In the originally published version of this manuscript, a potentially relevant study was inadvertently omitted. The citation for the study is as follows:

‘Rowe A, Onikpo F, Lama M, Deming M.S (2012). Evaluating health worker performance in Benin using the simulated client method with real children. Implementation Science. 7:95’

Upon investigation, the authors noted that while the above-mentioned study was retrieved by a literature search and picked up during the initial screening process, it was inadvertently excluded due to a single coding error within a large spreadsheet, which evaded subsequent scrutiny. The authors confirm that this was an isolated coding error with no significant impact to the findings and conclusions. To which end, the following revisions have been made to acknowledge the inclusion of the study in the review:

- The number of direct comparison studies included in the review is 13. It remains correct to say in Figure 1 that 384 records were potentially eligible to be included in the review based on inclusion criteria. However, 371 not 372 studies were excluded because methods were not directly compared and 13 not 12 studies in total are included in the review.
- A new row added to Table 1 to describe the study by Rowe *et al*. (2012). Table 1 is ordered alphabetically, so the new row appears between the rows for the studies by Pulford *et al.* (2014) and Tumlinson *et al.* (2014). The following information is included (starting horizontally from the left of the page, with each semi-colon in the list below denoting a new column): Rowe (2012), Benin; Management of child illnesses; 55 public (n = 47) and private (n = 8) health facilities; 89 health workers, trained and not trained in IMCI; 54 SP visits and 185 DO. A black dot appears in the DO + RE and SP columns. The IMCI abbreviation can be described as follows in the key at the bottom of the table: IMCI: Integrated Management of Childhood Illnesses. The main text is also amended as follows to describe this study: 10 out of 13 studies in Sub-Saharan Africa, five outpatient care services, five studies with children, around 3600 healthcare settings and 651 healthcare providers.
- Details about the study by Rowe *et al*. (2012) are added to the ‘Direct observation versus standardised patient’ comparisons; the second column of Table 4 (Standardised patients—Cons); the cost information in the ‘Descriptive comparisons between different methods;’ and under ‘Method preparation and implementation.’ It also added here that the Hawthorne effect was examined in five papers and quantified by three studies. Rowe *et al*. (2012) found that direct observation tended to show a higher percentage of quality indicator being fulfilled compared with standardised patients (range 1.7% lower to 61.1% higher, median difference 16.4% higher), which was suggestive of the Hawthorne effect. The observation was susceptible to confounding by different case mix and provider characteristics between the two sets (direct observation vs SP) of consultations. Sensitivity analysis excluding providers who were only assessed by one of the methods showed a smaller magnitude of difference between direct observation and SP (median 7.8%-points higher for direct observation).

