# Supplementary material for: Comparing the use of direct observation, standardized patients and exit interviews in low- and middle-income countries: a systematic review of methods of assessing quality of primary care
Source: Health Policy Plan. 2020 Dec 12;36(3):341–56. doi: 10.1093/heapol/czaa152 (PMC8058951; doi:10.1093/heapol/czaa152)
Supplement: czaa152_Supp [file czaa152_supp.zip › czaa152_Supplementary_Data.docx]

**APPENDIX**

| ***Direct comparison studies* – MEDLINE^a^** | |
| --- | --- |
| **#** | **Search Terms** |
| 1 | Developing Countries |
| 2 | (Africa or Asia or Caribbean or West Indies or South America or Latin America or Central America or Afghanistan or Albania or Algeria or Angola or Antigua or Barbuda or Argentina or Armenia or Armenian or Aruba or Azerbaijan or Bahrain or Bangladesh or Barbados or Benin or Byelarus or Byelorussian or Belarus or Belorussian or Belorussia or Belize or Bhutan or Bolivia or Bosnia or Herzegovina or Hercegovina or Botswana or Brasil or Brazil or Bulgaria or Burkina Faso or Burkina Fasso or Upper Volta or Burundi or Urundi or Cambodia or Khmer Republic or Kampuchea or Cameroon or Cameroons or Cameron or Camerons or Cape Verde or Central African Republic or Chad or Chile or China or Colombia or Comoros or Comoro Islands or Comores or Mayotte or Congo or Zaire or Costa Rica or Cote d'Ivoire or Ivory Coast or Croatia or Cuba or Cyprus or Czechoslovakia or Czech Republic or Slovakia or Slovak Republic or Djibouti or French Somaliland or Dominica or Dominican Republic or East Timor or East Timur or Timor Leste or Ecuador or Egypt or United Arab Republic or El Salvador or Eritrea or Estonia or Ethiopia or Fiji or Gabon or Gabonese Republic or Gambia or Gaza or Georgia Republic or Georgian Republic or Ghana or Gold Coast or Greece or Grenada or Guatemala or Guinea or Guam or Guiana or Guyana or Haiti or Honduras or Hungary or India or Maldives or Indonesia or Iran or Iraq or Isle of Man or Jamaica or Jordan or Kazakhstan or Kazakh or Kenya or Kiribati or Korea or Kosovo or Kyrgyzstan or Kirghizia or Kyrgyz Republic or Kirghiz or Kirgizstan or Lao PDR or Laos or Latvia or Lebanon or Lesotho or Basutoland or Liberia or Libya or Lithuania or Macedonia or Madagascar or Malagasy Republic or Malaysia or Malaya or Malay or Sabah or Sarawak or Malawi or Nyasaland or Mali or Malta or Marshall Islands or Mauritania or Mauritius or Agalega Islands or Mexico or Micronesia or Middle East or Moldova or Moldovia or Moldovian or Mongolia or Montenegro or Morocco or Ifni or Mozambique or Myanmar or Myanma or Burma or Namibia or Nepal or Netherlands Antilles or New Caledonia or Nicaragua or Niger or Nigeria or Northern Mariana Islands or Oman or Muscat or Pakistan or Palau or Palestine or Panama or Paraguay or Peru or Philippines or Philipines or Phillipines or Phillippines or Poland or Portugal or Puerto Rico or Romania or Rumania or Roumania or Russia or Russian or Rwanda or Ruanda or Saint Kitts or St Kitts or Nevis or Saint Lucia or St Lucia or Saint Vincent or St Vincent or Grenadines or Samoa or Samoan Islands or Navigator Island or Navigator Islands or Sao Tome or Saudi Arabia or Senegal or Serbia or Montenegro or Seychelles or Sierra Leone or Slovenia or Sri Lanka or Ceylon or Solomon Islands or Somalia or South Africa or Sudan or Suriname or Surinam or Swaziland or Syria or Tajikistan or Tadzhikistan or Tadjikistan or Tadzhik or Tanzania or Thailand or Togo or Togolese Republic or Tonga or Trinidad or Tobago or Tunisia or Turkey or Turkmenistan or Turkmen or Uganda or Ukraine or Uruguay or USSR or Soviet Union or Union of Soviet Socialist Republics or Uzbekistan or Uzbek or Vanuatu or New Hebrides or Venezuela or Vietnam or Viet Nam or West Bank or Yemen or Yugoslavia or Zambia or Zimbabwe or Rhodesia) |
| 2 | ((developing or less* developed or under developed or underdeveloped or middle income or low* income or underserved or under served or deprived or poor*) adj (countr* or nation? or population? or world)) |
| 3 | ((developing or less* developed or under developed or underdeveloped or middle income or low* income) adj (economy or economies)) |
| 4 | (low* adj (gdp or gnp or gross domestic or gross national)) |
| 5 | (low adj3 middle adj3 countr*) |
| 6 | (lmic or lmics or third world or lami countr*) |
| 7 | transitional countr* |
| 8 | or/1-7 |
| 9 | (Quality ADJ3 (care OR healthcare OR health care)) |
| 10 | (Standard ADJ3 (care OR healthcare OR health care)) |
| 11 | Clinical competence/ |
| 12 | Professional competence/ |
| 13 | Quality assurance, healthcare/ |
| 14 | Quality indicators, healthcare/ |
| 15 | Simulat* patient? OR Standardi?ed patient? |
| 16 | Mystery shop* |
| 17 | Direct observation? |
| 18 | (Observed ADJ3 (doctor consultation? or consultation? or doctor interaction? or interaction?)) |
| 19 | (Observed ADJ3 (patient encounter? or patient consultation? or patient interaction?)) |
| 20 | (Video* ADJ3 observation) |
| 21 | (Audio* ADJ3 observation) |
| 22 | (Exit ADJ3 (interview* or questionnaire? or survey?)) |
| 23 | (Debrief* ADJ3 (patient? or medical or clinical)) |
| 24 | 9 OR 10 OR 11 OR 12 OR 13 OR 14 |
| 25 | 15 OR 16 OR 17 OR 18 OR 19 OR 20 OR 21 OR 22 OR 23 |
| 26 | 8 AND 24 AND 25 |

^a^ An adapted version of this search was used in CINAHL, Cochrane Library and ASSIA databases.

| ***Direct comparison studies* - PSYCINFO** | |
| --- | --- |
| **#** | **Search Terms** |
| 1 | Developing Countries |
| 2 | (Africa or Asia or Caribbean or West Indies or South America or Latin America or Central America or Afghanistan or Albania or Algeria or Angola or Antigua or Barbuda or Argentina or Armenia or Armenian or Aruba or  Azerbaijan or Bahrain or Bangladesh or Barbados or Benin or Byelarus or Byelorussian or Belarus or Belorussian or  Belorussia or Belize or Bhutan or Bolivia or Bosnia or Herzegovina or Hercegovina or Botswana or Brasil or Brazil or  Bulgaria or Burkina Faso or Burkina Fasso or Upper Volta or Burundi or Urundi or Cambodia or Khmer Republic or Kampuchea  or Cameroon or Cameroons or Cameron or Camerons or Cape Verde or Central African Republic or Chad or Chile or China or  Colombia or Comoros or Comoro Islands or Comores or Mayotte or Congo or Zaire or Costa Rica or Cote d'Ivoire or Ivory Coast  or Croatia or Cuba or Cyprus or Czechoslovakia or Czech Republic or Slovakia or Slovak Republic or Djibouti or French  Somaliland or Dominica or Dominican Republic or East Timor or East Timur or Timor Leste or Ecuador or Egypt or United Arab Republic or El Salvador or Eritrea or Estonia or Ethiopia or Fiji or Gabon or  Gabonese Republic or Gambia or Gaza or Georgia Republic or Georgian Republic or Ghana or Gold Coast or Greece or Grenada  or Guatemala or Guinea or Guam or Guiana or Guyana or Haiti or Honduras or Hungary or India or Maldives or Indonesia or  Iran or Iraq or Isle of Man or Jamaica or Jordan or Kazakhstan or Kazakh or Kenya or Kiribati or Korea or Kosovo or Kyrgyzstan or Kirghizia or  Kyrgyz Republic or Kirghiz or Kirgizstan or Lao PDR or Laos or Latvia or Lebanon or Lesotho or Basutoland or Liberia or  Libya or Lithuania or Macedonia or Madagascar or Malagasy Republic or Malaysia or Malaya or Malay or Sabah or Sarawak or  Malawi or Nyasaland or Mali or Malta or Marshall Islands or Mauritania or Mauritius or Agalega Islands or Mexico or  Micronesia or Middle East or Moldova or Moldovia or Moldovian or Ecuador or Egypt or United Arab Republic or El Salvador or Eritrea or Estonia or Ethiopia or Fiji or Gabon or  Gabonese Republic or Gambia or Gaza or Georgia Republic or Georgian Republic or Ghana or Gold Coast or Greece or Grenada  or Guatemala or Guinea or Guam or Guiana or Guyana or Haiti or Honduras or Hungary or India or Maldives or Indonesia or  Iran or Iraq or Isle of Man or Mongolia or Montenegro or Morocco or Ifni or Mozambique or Myanmar or Myanma or Burma or Namibia or Nepal or  Netherlands Antilles or New Caledonia or Nicaragua or Niger or Nigeria or Northern Mariana Islands or Oman or Muscat or  Pakistan or Palau or Palestine or Panama or Paraguay or Peru or Philippines or Philipines or Phillipines or Phillippines  or Poland or Portugal or Puerto Rico or Romania or Rumania or Roumania or Russia or Russian or Rwanda or Ruanda or Saint Kitts or St Kitts or Nevis or Saint Lucia or St Lucia or Saint Vincent or St Vincent or Grenadines or Samoa  or Samoan Islands or Navigator Island or Navigator Islands or Sao Tome or Saudi Arabia or Senegal or Serbia or  Montenegro or Seychelles or Sierra Leone or Slovenia or Sri Lanka or Ceylon or Solomon Islands or Somalia or South  Africa or Sudan or Suriname or Surinam or Swaziland or Syria or Tajikistan or Tadzhikistan or Tadjikistan or Tadzhik or Tanzania or Thailand or Togo or Togolese Republic or Tonga or Trinidad or Tobago or Tunisia or Turkey  or Turkmenistan or Turkmen or Uganda or Ukraine or Uruguay or USSR or Soviet Union or Union of Soviet Socialist  Republics or Uzbekistan or Uzbek or Vanuatu or New Hebrides or Venezuela or Vietnam or Viet Nam or West Bank or Yemen or  Yugoslavia or Zambia or Zimbabwe or Rhodesia |
| 2 | ((developing or less* developed or under developed or underdeveloped or middle income or low* income or  underserved or under served or deprived or poor*) adj (countr* or nation? or population? or world)) |
| 3 | ((developing or less* developed or under developed or underdeveloped or middle income or low* income) adj (economy or economies)) |
| 4 | (low* adj (gdp or gnp or gross domestic or gross national)) |
| 5 | (low adj3 middle adj3 countr*) |
| 6 | (lmic or lmics or third world or lami countr* |
| 7 | transitional countr* |
| 8 | or/1-7 |
| 9 | (Quality ADJ3 (care OR healthcare OR health care)) |
| 10 | (Standard ADJ3 (care OR healthcare OR health care)) |
| 11 | Clinical competence |
| 12 | Technical competence |
| 13 | EXP Quality of care |
| 14 | Quality indicators, healthcare/ |
| 15 | Simulat* patient? OR Standardi?ed patient? |
| 16 | Mystery shop* |
| 17 | Direct observation? |
| 18 | (Observed ADJ3 (doctor consultation? or consultation? or interaction? or doctor interaction?)) |
| 19 | (Observed ADJ3 (patient encounter? or patient consultation? or patient interaction?)) |
| 20 | (Video* ADJ3 observation) |
| 21 | (Audio* ADJ3 observation) |
| 22 | (Exit ADJ3 (interview* or questionnaire? or survey?)) |
| 23 | (Debrief* ADJ3 (patient? or medical or clinical)) |
| 24 | 9 OR 10 OR 11 OR 12 OR 13 OR 14 |
| 25 | 15 OR 16 OR 17 OR 18 OR 19 OR 20 OR 21 OR 22 OR 23 |
| 26 | 8 AND 24 AND 25 |

| ***Direct comparison studies* - EMBASE** | |
| --- | --- |
| **#** | **Search Terms** |
| 1 | Developing Country |
| 2 | (Africa or Asia or Caribbean or West Indies or South America or Latin America or Central America or Afghanistan or Albania or Algeria or Angola or Antigua or Barbuda or Argentina or Armenia or Armenian or Aruba or  Azerbaijan or Bahrain or Bangladesh or Barbados or Benin or Byelarus or Byelorussian or Belarus or Belorussian or  Belorussia or Belize or Bhutan or Bolivia or Bosnia or Herzegovina or Hercegovina or Botswana or Brasil or Brazil or  Bulgaria or Burkina Faso or Burkina Fasso or Upper Volta or Burundi or Urundi or Cambodia or Khmer Republic or Kampuchea  or Cameroon or Cameroons or Cameron or Camerons or Cape Verde or Central African Republic or Chad or Chile or China or  Colombia or Comoros or Comoro Islands or Comores or Mayotte or Congo or Zaire or Costa Rica or Cote  d'Ivoire Ivory Coast or Croatia or Cuba or Cyprus or Czechoslovakia or Czech Republic or Slovakia or Slovak Republic or Djibouti or French Somaliland or Dominica or Dominican Republic or East Timor or East Timur or Timor Leste or Ecuador or  Egypt or United Arab Republic or El Salvador or Eritrea or Estonia or Ethiopia or Fiji or Gabon or Gabonese Republic or  Gambia or Gaza or Georgia Republic or Georgian Republic or Ghana or Gold Coast or Greece or Grenada or Guatemala or Guinea or Guam or Guiana or Guyana or Haiti or Honduras or Hungary or India or  Maldives or Indonesia or Iran or Iraq or Isle of Man or Jamaica or Jordan or Kazakhstan or Kazakh or Kenya or Kiribati  or Korea or Kosovo or Kyrgyzstan or Kirghizia or Kyrgyz Republic or Kirghiz or Kirgizstan or Lao PDR or Laos or Latvia  or Lebanon or Lesotho or Basutoland or Liberia or Libya or Lithuania or Macedonia or Madagascar or Malagasy Republic or Malaysia or Malaya or Malay or Sabah or Sarawak or Malawi or  Nyasaland or Mali or Malta or Marshall Islands or Mauritania or Mauritius or Agalega Islands or Mexico or Micronesia or  Middle East or Moldova or Moldovia or Moldovian or Mongolia or Montenegro or Morocco or Ifni or Mozambique or Myanmar or  Myanma or Burma or Namibia or Nepal or Netherlands Antilles or New Caledonia or Nicaragua or Niger or Nigeria or  Northern Mariana Islands or Oman or Muscat or Pakistan or Palau or Palestine or Panama or Paraguay or Peru or Philippines or Philipines or Phillipines or Phillippines or Poland or  Portugal or Puerto Rico or Romania or Rumania or Roumania or Russia or Russian or Rwanda or Ruanda or Saint Kitts or St  Kitts or Nevis or Saint Lucia or St Lucia or Saint Vincent or St Vincent or Grenadines or Samoa or Samoan Islands or  Navigator Island or Navigator Islands or Sao Tome or Saudi Arabia or Senegal or Serbia or Montenegro or Seychelles or  Sierra Leone or Slovenia or Sri Lanka or Ceylon or Solomon Islands or Somalia or South Africa or Sudan or Suriname or Surinam or Swaziland or Syria or Tajikistan or Tadzhikistan or  Tadjikistan or Tadzhik or Tanzania or Thailand or Togo or Togolese Republic or Tonga or Trinidad or Tobago or Tunisia or  Turkey or Turkmenistan or Turkmen or Uganda or Ukraine or Uruguay or USSR or Soviet Union or Union of Soviet Socialist Republics or Uzbekistan or Uzbek or Vanuatu or New Hebrides or  Venezuela or Vietnam or Viet Nam or West Bank or Yemen or Yugoslavia or Zambia or Zimbabwe or Rhodesia |
| 3 | ((developing or less* developed or under developed or underdeveloped or middle income or low* income or  underserved or under served or deprived or poor*) adj (countr* or nation? or population? or world)).ti,ab. |
| 4 | ((developing or less* developed or under developed or underdeveloped or middle income or low* income) adj  (economy or economies)).ti,ab. |
| 5 | (low* adj (gdp or gnp or gross domestic or gross national)).ti,ab. |
| 6 | (low adj3 middle adj3 countr*).ti,ab. |
| 7 | (lmic or lmics or third world or lami countr*).ti,ab. |
| 8 | transitional countr*.ti,ab. |
| 9 | or/1-8 |
| 10 | (Quality ADJ3 (care OR healthcare OR health care)) |
| 11 | (Standard ADJ3 (care OR healthcare OR health care)) |
| 12 | Clinical competence/ |
| 13 | Technical competence |
| 14 | Health care quality/ |
| 15 | Simulat* patient? OR Standardi?ed patient? |
| 16 | Mystery shop* |
| 17 | Direct observation? |
| 18 | (Observed ADJ3 (doctor consultation? or consultation? or interaction? or doctor interaction?)) |
| 19 | (Observed ADJ3 (patient encounter? or patient consultation? or patient interaction?)) |
| 20 | (Video* ADJ3 observation) |
| 21 | (Audio* ADJ3 observation) |
| 22 | (Exit ADJ3 (interview* or questionnaire? or survey?)) |
| 23 | (Debrief* ADJ3 (patient? or medical or clinical)) |
| 24 | 10 OR 11 OR 12 OR 13 OR 14 |
| 25 | 15 OR 16 OR 17 OR 18 OR 19 OR 20 OR 21 OR 22 OR 23 |
| 26 | 9 AND 24 AND 25 |

| ***Overview of Reviews* - MEDLINE** | |
| --- | --- |
| **#** | **Search Terms** |
| 1 | (quality ADJ3 (care OR healthcare OR health care)) |
| 2 | (standard ADJ3 (care OR healthcare OR health care)) |
| 3 | Peer review, healthcare/ |
| 4 | Process assessment (healthcare)/ |
| 5 | Simulat* patient? OR Standardi?ed patient? |
| 6 | Observation? |
| 7 | Interview* |
| 8 | Surveys and Questionnaires/ |
| 9 | Vignette? |
| 10 | Repeat* ADJ3 exam* |
| 11 | Second opinion? |
| 12 | Valid* |
| 13 | Feasib* |
| 14 | Reliab* |
| 15 | Exp Costs and costs analysis/ |
| 16 | (Measur*OR Assess* OR Apprais* OR Evaluat* ADJ3 (quality or standard)) |
| 17 | Review literature as topic/ |
| 18 | Review/ |
| 19 | Systematic review |
| 20 | Narrative review |
| 21 | 1 OR 2 OR 3 OR 4 |
| 22 | 5 OR 6 OR 7 OR 8 OR 9 OR 10 OR 11 |
| 23 | 12 OR 13 OR 14 OR 15 OR 16 |
| 24 | 17 OR 18 OR 19 OR 20 |
| 25 | 21 AND 22 AND 23 AND 24 |

| ***Overview of Reviews* - PSYCINFO** | |
| --- | --- |
| **#** | **Search Terms** |
| 1 | (quality ADJ3 (care OR healthcare OR health care)) |
| 2 | (standard ADJ3 (care OR healthcare OR health care)) |
| 3 | Simulat* patient? OR Standardi?ed patient? |
| 4 | Observation? |
| 5 | Interview* |
| 6 | Surveys and Questionnaires/ |
| 7 | Vignette? |
| 8 | Repeat* ADJ3 exam* |
| 9 | Second opinion? |
| 10 | Valid* |
| 11 | Feasib* |
| 12 | Reliab* |
| 13 | Exp Costs and costs analysis/ |
| 14 | (Measur*OR Assess* OR Apprais* OR Evaluat* ADJ3 (quality or standard)) |
| 15 | Review literature as topic/ |
| 16 | Review/ |
| 17 | Systematic review |
| 18 | Narrative review |
| 19 | 1 OR 2 |
| 20 | 3 OR 4 OR 5 OR 6 OR 7 OR 8 OR 9 |
| 21 | 10 OR 11 OR 12 OR 13 OR 14 |
| 22 | 15 OR 16 OR 17 OR 18 |
| 23 | 19 AND 20 AND 21 AND 22 |

| ***Overview of Reviews* - EMBASE** | |
| --- | --- |
| **#** | **Search Terms** |
| 1 | (quality ADJ3 (care OR healthcare OR health care)) |
| 2 | (standard ADJ3 (care OR healthcare OR health care)) |
| 3 | Simulat* patient? OR Standardi?ed patient? |
| 4 | Observation/ |
| 5 | Interview/ |
| 6 | Surveys and Questionnaires |
| 7 | Vignette/ |
| 8 | Repeat* ADJ3 exam* |
| 9 | Second opinion? |
| 10 | Valid* |
| 11 | Feasib* |
| 12 | Reliability/ |
| 13 | Costs and costs analysis |
| 14 | (Measur*OR Assess* OR Apprais* OR Evaluat* ADJ3 (quality or standard)) |
| 15 | Review |
| 16 | Systematic review |
| 17 | Systematic review (topic) |
| 18 | Narrative review |
| 19 | 1 OR 2 |
| 20 | 3 OR 4 OR 5 OR 6 OR 7 OR 8 OR 9 |
| 21 | 10 OR 11 OR 12 OR 13 OR 14 |
| 22 | 15 OR 16 OR 17 OR 18 |
| 23 | 19 AND 20 AND 21 AND 22 |

**Appendix Table 1. Performance of direct observation against other reference standards**

|  |  |  |  | **Performance of the assessment method vs reference standard** | | | | | | | | |
| --- | --- | --- | --- | --- | --- | --- | --- | --- | --- | --- | --- | --- |
| **Quality item /indicator** | **Study** | **Comparison* (assessment method vs reference standard)** | **Provider performance based on reference standard** | **Agreement (%)** | **Kappa** | **Sensitivity (%)** | **Specificity (%)** | **PPV (%)** | **NPV (%)** | **Area under ROC curve** | **LR+** | **LR-** |
| **Childhood illnesses -general** |  |  |  |  |  |  |  |  |  |  |  |  |
| Correct management of major iCCM illnesses ^a^ | Miller et al. (2015) | DO vs DO + RE | 63  (56 to 70) | - | - | 97 | 59 | - | - | 0.78 | - | - |
| Correct management of severe illness | Miller et al. (2015) | DO vs DO + RE | 32  (20 to 48) | - | - | 83 | 48 | - | - | 0.66 | - | - |
| Correct dose, duration, and schedule for all treatments | Miller et al. (2015) | DO vs DO + RE | 92  (87 to 96) | - | - | 100 | 100 | - | - | 1.00 | - | - |
| Unnecessary antibiotic or antimalarial prescribed | Miller et al. (2015) | DO vs DO + RE | 5  (3 to 10) | - | - | 39 | 99.6 | - | - | 0.69 | - | - |
| Correct referral | Miller et al. (2015) | DO vs DO + RE | 53  (39 to 66) | - | - | 94 | 14 | - | - | 0.54 | - | - |
| **Childhood illness – acute respiratory illness** |  |  |  |  |  |  |  |  |  |  |  |  |
| Counted child’s respiratory rate | Hermida et al. (1999) | DO vs DO (exp) | 37 | - | - | 100 ^b^ | 72 ^b^ | - | - | - | - | - |
| Correct classification of fast breathing – uncomplicated illness | Cardemil et al. (2012) | DO vs DO + RE | 15  (11 to 19) | 77 | 0.35  (0.23 to 0.47) | 59  (46 to 72) | 82  (75 to 88) | - | - | - | - | - |
| Correct classification of fast breathing – severe illness | Cardemil et al. (2012) | DO vs DO + RE | 5 (3 to 7) | 81 | 0.53  (0.31 to 0.76) | 68  (41 to 95) | 86  (75 to 96) | - | - | - | - | - |
| Correct management of fast breathing – uncomplicated illness | Cardemil et al. (2012) | DO vs DO + RE | - | 73 | 0.31  (0.19 to 0.43) | 63  (50 to 77) | 75  (68 to 83) | - | - | - | - | - |
| Correct treatment of pneumonia | Miller et al. (2015) | DO vs DO + RE | 73  (57 to 85) | - | - | 100 | 30 | - | - | 0.65 | - | - |
| Correct management of fast breathing – severe illness | Cardemil et al. (2012) | DO vs DO + RE | - | 45 | -0.15  (-0.38 to 0.07) | 32  (3 to 60) | 51  (35 to 67) | - | - | - | - | - |
| Prescribed antibiotic | Hermida et al. (1999) | DO vs DO (exp) | - | - | - | 93 ^b^ | 78 ^b^ | - | - | - | - | - |
| Advised mother on recognising danger signs for pneumonia | Hermida et al. (1999) | DO vs DO (exp) | 10 | - | - | 100 | 79 | - | - | - | - | - |
| **Childhood illness - diarrhoea** |  |  |  |  |  |  |  |  |  |  |  |  |
| Asked about blood in faeces | Hermida et al. (1999) | DO vs DO (exp) | 32 | - | - | 83 ^b^ | 84 ^b^ | - | - | - | - | - |
| Evaluated mouth dryness | Hermida et al. (1999) | DO vs DO (exp) | 32 | - | - | 94 ^b^ | 39 ^b^ | - | - | - | - | - |
| Correct classification of diarrhoea – uncomplicated illness | Cardemil et al. (2012) | DO vs DO + RE | 25  (21 to 29) | 97 | 0.92  (0.88 to 0.97) | 92  (86 to 97) | 99  (98 to 100) | - | - | - | - | - |
| Correct classification of diarrhoea – severe illness | Cardemil et al. (2012) | DO vs DO + RE | 4  (2 to 6) | 79 | 0.40  (0.14 to 0.66) | 57  (26 to 89) | 85  (74 to 96) | - | - | - | - | - |
| Correct management of diarrhoea – uncomplicated illness | Cardemil et al. (2012) | DO vs DO + RE | - | 96 | 0.91  (0.86 to 0.97) | 90  (82 to 98) | 99  (98 to 100) | - | - | - | - | - |
| Correct management of diarrhoea – severe illness | Cardemil et al. (2012) | DO vs DO + RE | - | 43 | -0.05  (-0.24 to 0.15) | 50  (12 to 88) | 42  (23 to 61) | - | - | - | - | - |
| Correct treatment of diarrhoea | Miller et al. (2015) | DO vs DO + RE | 79  (71 to 85) | - | - | 90 | 91 | - | - | 0.90 | - | - |
| Prescribed oral rehydration solution | Hermida et al. (1999) | DO vs DO (exp) | 68 | - | - | 92 ^b^ | 72 ^b^ | - | - | - | - | - |
| **Childhood illness - fever** |  |  |  |  |  |  |  |  |  |  |  |  |
| Correct classification of fever – uncomplicated illness | Cardemil et al. (2012) | DO vs DO + RE | 63  (58 to 69) | 95 | 0.86  (0.79 to 0.92) | 93  (90 to 97) | 99  (96 to 100) | - | - | - | - | - |
| Correct classification of fever – severe illness | Cardemil et al. (2012) | DO vs DO + RE | 13  (10 to 17) | 88 | 0.72  (0.54 to 0.90) | 86  (76 to 96) | 94  (82 to 100) | - | - | - | - | - |
| Correct management of fever – uncomplicated illness | Cardemil et al. (2012) | DO vs DO + RE | - | 89 | 0.70  (0.60 to 0.79) | 87  (81 to 92) | 98  (94 to 100) | - | - | - | - | - |
| Correct management of fever – severe illness | Cardemil et al. (2012) | DO vs DO + RE | - | 44 | 0.01  (-0.18 to 0.19) | 36  (22 to 51) | 65  (38 to 91) | - | - | - | - | - |
| **Malnutrition in children** |  |  |  |  |  |  |  |  |  |  |  |  |
| Correct treatment of malnutrition | Miller et al. (2015) | DO vs DO + RE | 61  (42 to 78) | - | - | 37 | 75 | - | - | 0.56 | - | - |
| Vitamin A supplement given when needed | Miller et al. (2015) | DO vs DO + RE | 19  (10 to 32) | - | - | 92 | 56 | - | - | 0.74 | - | - |
| **Other** |  |  |  |  |  |  |  |  |  |  |  |  |
| Mebendazole given when needed (childhood worm infection) | Miller et al. (2015) | DO vs DO + RE | 21  (9 to 40) | - | - | 83 | 65 | - | - | 0.74 | - | - |
| Advised mother when to continue breastfeeding | Hermida et al. (1999) | DO vs DO (exp) | 30 | - | - | 20 ^b^ | 87 ^b^ | - | - | - | - | - |
| Counselled mother on family planning | Hermida et al. (1999) | DO vs DO (exp) | 21 | - | - | 86 ^b^ | 98 ^b^ | - | - | - | - | - |
| **Case management for sexually transmitted diseases** |  |  |  |  |  |  |  |  |  |  |  |  |
| Asked about onset of symptoms | Franco et al. (1997) | DO ^c^ vs SP | 65 | 55 | -0.18 | - | - | - | - | - | - | - |
| Asked about onset of symptoms | Franco et al. (1997) | DO ^d^ vs SP | 65 | 65 | 0.21 | - | - | - | - | - | - | - |
| Asked about contact with high-risk partners | Franco et al. (1997) | DO ^c^ vs SP | 35 | 60 | 0.27 | - | - | - | - | - | - | - |
| Asked about contact with high-risk partners | Franco et al. (1997) | DO ^d^ vs SP | 35 | 55 | -0.10 | - | - | - | - | - | - | - |
| Asked about recent new sexual partners | Franco et al. (1997) | DO ^c^ vs SP | 11 | 56 | 0.16 | - | - | - | - | - | - | - |
| Asked about recent new sexual partners | Franco et al. (1997) | DO ^d^ vs SP | 11 | 84 | 0.50 | - | - | - | - | - | - | - |
| Carried out any physical examination | Franco et al. (1997) | DO ^c^ vs SP | 37 | 58 | 0.23 | - | - | - | - | - | - | - |
| Carried out any physical examination | Franco et al. (1997) | DO ^d^ vs SP | 37 | 63 | 0.27 | - | - | - | - | - | - | - |
| Exposed patient fully | Franco et al. (1997) | DO ^c^ vs SP | 43 | 43 | -0.12 | - | - | - | - | - | - | - |
| Exposed patient fully | Franco et al. (1997) | DO ^d^ vs SP | 43 | 57 | 0.13 | - | - | - | - | - | - | - |
| Retracted foreskin (male patients) | Franco et al. (1997) | DO ^c^ vs SP | 43 | NA | NA | - | - | - | - | - | - | - |
| Retracted foreskin (male patients) | Franco et al. (1997) | DO ^d^ vs SP | 43 | NA | NA | - | - | - | - | - | - | - |
| Gave correct drugs (syndromic management for urethral discharge) | Franco et al. (1997) | DO ^c^ vs SP | 19 | 69 | 0.26 | - | - | - | - | - | - | - |
| Gave correct drugs (syndromic management for urethral discharge | Franco et al. (1997) | DO ^d^ vs SP | 19 | 75 | 0.18 | - | - | - | - | - | - | - |
| Gave correct drugs/ dosages for urethral discharge | Franco et al. (1997) | DO ^c^ vs SP | 0 | NA | NA | - | - | - | - | - | - | - |
| Gave correct drugs/ dosages for urethral discharge | Franco et al. (1997) | DO ^d^ vs SP | 0 | NA | NA | - | - | - | - | - | - | - |
| Advised to finish treatment | Franco et al. (1997) | DO ^c^ vs SP | 24 | 53 | 0.23 | - | - | - | - | - | - | - |
| Advised to finish treatment | Franco et al. (1997) | DO ^d^ vs SP | 24 | 65 | 0.27 | - | - | - | - | - | - | - |
| Advised to use condoms | Franco et al. (1997) | DO ^c^ vs SP | 20 | 50 | 0.00 | - | - | - | - | - | - | - |
| Advised to use condoms | Franco et al. (1997) | DO ^d^ vs SP | 20 | 80 | 0.38 | - | - | - | - | - | - | - |
| Gave instructions on how to use condom | Franco et al. (1997) | DO ^c^ vs SP | 0 | NA | NA | - | - | - | - | - | - | - |
| Gave instructions on how to use condom | Franco et al. (1997) | DO ^d^ vs SP | 0 | NA | NA | - | - | - | - | - | - | - |
| Told to have partner treated | Franco et al. (1997) | DO ^c^ vs SP | 32 | 47 | 0.16 | - | - | - | - | - | - | - |
| Told to have partner treated | Franco et al. (1997) | DO ^d^ vs SP | 32 | 47 | -0.03 | - | - | - | - | - | - | - |
| Gave or prescribed condoms to patient | Franco et al. (1997) | DO ^c^ vs SP | 6 | 75 | -0.10 | - | - | - | - | - | - | - |
| Gave or prescribed condoms to patient | Franco et al. (1997) | DO ^d^ vs SP | 6 | 81 | -0.09 | - | - | - | - | - | - | - |
| **Family planning** |  |  |  |  |  |  |  |  |  |  |  |  |
| Discussed 2 or more methods with client | Tumlinson et al. (2014) | DO vs SP | 95 | 84 (70 to 93) | - | 88 (74 to 96) | NA | 95 (83 to 99) | NA | - | NA | NA |
| Asked client about her preferred method | Tumlinson et al. (2014) | DO vs SP | 98 | 95 (85 to 99) | - | 98 (88 to 100) | NA | 98 (88 to 100) | NA |  | NA | NA |
| Helped client select a method | Tumlinson et al. (2014) | DO vs SP | 64 | 61 (46 to 76) | - | 96 (82 to 99) | 0 (0 to 21) | 63 (47 to 77) | NA |  | 1.0 | NA |
| Discussed side effects | Tumlinson et al. (2014) | DO vs SP | 66 | 64 (48 to 78) | - | 83 (64 to 94) | 27 (8 to 55) | 69 (51 to 83) | 44 (14 to 79) |  | 1.1 | 0.6 |
| Discussed management of side effects | Tumlinson et al. (2014) | DO vs SP | 45 | 52 (37 to 68) | - | 55 (32 to 77) | 50 (29 to 71) | 48 (27 to 69) | 57 (34 to 78) |  | 1.1 | 0.9 |
| Discussed warning signs | Tumlinson et al. (2014) | DO vs SP | 2 | 95 (85 to 99) | - | NA | 95 (84 to 99) | NA | 100 (91 to 100) |  | NA | NA |
| Discussed what to do if warning signs occur | Tumlinson et al. (2014) | DO vs SP | 2 | 95 (85 to 99) | - | NA | 95 (84 to 99) | NA | 100 (91 to 100) |  | NA | NA |
| Told client how to use selected method | Tumlinson et al. (2014) | DO vs SP | 73 | 59 (43 to 74) | - | 72 (53 to 86) | 25 (6 to 57) | 72 (53 to 86) | 25 (6 to 57) |  | 1.0 | 1.1 |
| Treat client with respect | Tumlinson et al. (2014) | DO vs SP | 89 | 89 (75 to 96) | - | 100 (91 to 100) | NA | 89 (75 to 96) | NA |  | NA | NA |
| Asked client about her reproductive goals | Tumlinson et al. (2014) | DO vs SP | 2 | 57 (41 to 72) | - | NA | 58 (42 to 73) | 0 (0 to 19) | 96 (80 to 100) |  | NA | NA |
| Asked client whether she has any questions | Tumlinson et al. (2014) | DO vs SP | 34 | 45 (30 to 61) | - | 60 (32 to 84) | 38 (21 to 58) | 33 (17 to 54) | 65 (38 to 86) |  | 1.0 | 1.1 |
| Took client’s medical history | Tumlinson et al. (2014) | DO vs SP | 12 | 86 (72 to 95) | - | NA | 92 (79 to 98) | NA | 92 (79 to 98) |  | NA | NA |
| Told client when to return for resupply/ follow-up | Tumlinson et al. (2014) | DO vs SP | 79 | 63 (47 to 77) | - | 74 (56 to 87) | 22 (3 to 60) | 78 (60 to 91) | 18 (2 to 52) |  | 0.9 | 1.2 |
| Gave client an appointment/ reminder card | Tumlinson et al. (2014) | DO vs SP | 36 | 39 (24 to 55) | - | 56 (30 to 80) | 29 (13 to 49) | 31 (15 to 51) | 53 (27 to 79) |  | 0.8 | 1.5 |
| Told client what to do if she experiences problems | Tumlinson et al. (2014) | DO vs SP | 27 | 37 (22 to 53) | - | 73 (39 to 94) | 23 (10 to 42) | 26 (12 to 45) | 70 (35 to 93) |  | 0.9 | 1.2 |
| Told client where to go for resupply | Tumlinson et al. (2014) | DO vs SP | 64 | 57 (29 to 82) | - | 78 (40 to 97) | NA | 64 (31 to 89) | NA |  | NA | NA |
| Offered client additional services | Tumlinson et al. (2014) | DO vs SP | 11 | 41 (26 to 57) | - | NA | 38 (23 to 55) | 11 (2 to 29) | 88 (64 to 99) |  | NA | NA |

Numbers provided in curly brackets are confidence intervals. These are shown on when they were reported by original authors

*Comparison: DO: direct observation; DO + RE: direct observation plus re-examination; DO (exp): direct observation undertaken by specially trained, experienced observer; SP: standardised patient

^a^ iCCM: integrated community case management of childhood illness. Major iCCM illnesses include diarrhoea, malaria, measles, malnutrition, and danger signs

^b^ Sensitivity and specificity were defined in an opposite way in this study (e.g. sensitivity was defined as performance failures detected by the assessment compared with reference standard) compared with the definitions adopted in this review as described in the footnotes above; figures presented in this table have been inverted on this basis to reflect the standard definitions adopted in this review

^c^ For direct observation, a positive response was recorded if the provider was observed to have carried out the task on at least one patient

^d^ For direct observation, a positive response was recorded only if the provider was observed to have carried out the task on every patient

NA: not applicable (not evaluated) or not available (e.g. could not be calculated due to small numbers). -: not reported

**Appendix Table 2. Performance of patient/carer exit interview against direct observation and standardised patient**

|  |  |  |  | **Performance of the assessment method vs reference standard** | | | | | | | | |
| --- | --- | --- | --- | --- | --- | --- | --- | --- | --- | --- | --- | --- |
| **Quality item /indicator** | **Study** | **Comparison (assessment method vs reference standard)** | **Provider performance based on reference standard** | **Agreement (%)** | **Kappa** | **Sensitivity (%)** | **Specificity (%)** | **PPV (%)** | **NPV (%)** | **Area under ROC curve** | **LR+** | **LR-** |
| **Common illnesses** |  |  |  |  |  |  |  |  |  |  |  |  |
| All items (covering history taking questions, physical examination procedures, manner, general health education and referrals) | Leonard & Masatu (2006) | EX (pt) vs DO | 57 | 65 | 0.28 ^a^ | 72 ^b^ | 56 ^b^ | 68 ^b^ | 60 ^b^ | - | 1.6 ^b^ | 0.5 ^b^ |
| All items (same as above, but including an item-level random effect) | Leonard & Masatu (2006) | EX (pt) vs DO | - | - | 0.170 (SE 0.024) ^a^ | - | - | - | - | - | - | - |
| Clinician manner | Leonard & Masatu (2006) | EX (pt) vs DO | 77 | 77 | - | 100 ^b^ | 0 ^b^ | 77 ^b^ | NA | - | 1.0 ^b^ | NA |
| History taking | Leonard & Masatu (2006) | EX (pt) vs DO | 60 | 63 | - | 65 ^b^ | 60 ^b^ | 71 ^b^ | 53 ^b^ | - | 1.6 ^b^ | 0.6 ^b^ |
| Physical examination | Leonard & Masatu (2006) | EX (pt) vs DO | 39 | 69 | - | 64 ^b^ | 72 ^b^ | 60 ^b^ | 76 ^b^ | - | 2.3 ^b^ | 0.5 ^b^ |
| Health education | Leonard & Masatu (2006) | EX (pt) vs DO | 49 | 57 | - | 55 ^b^ | 59 ^b^ | 56 ^b^ | 58 ^b^ | - | 1.3 ^b^ | 0.8 ^b^ |
| **Acute childhood illnesses** |  |  |  |  |  |  |  |  |  |  |  |  |
| Asked about blood in faeces | Hermida et al. (1999) | EX (pt) vs DO (exp) | 32 | - | - | 89 | 87 | - | - | - | - | - |
| Evaluated mouth dryness | Hermida et al. (1999) | EX (pt) vs DO (exp) | 32 | - | - | NA | NA | - | - | - | - | - |
| Counted child’s respiratory rate | Hermida et al. (1999) | EX (pt) vs DO (exp) | 37 | - | - | 93 | 57 | - | - | - | - | - |
| Prescribed oral rehydration solution | Hermida et al. (1999) | EX (pt) vs DO (exp) | 68 | - | - | 97 | 78 | - | - | - | - | - |
| Prescribed antibiotic | Hermida et al. (1999) | EX (pt) vs DO (exp) | - | - | - | 82 | 78 | - | - | - | - | - |
| Advised mother when to continue breastfeeding | Hermida et al. (1999) | EX (pt) vs DO (exp) | 30 | - | - | 40 | 70 | - | - | - | - | - |
| Advised mother on recognising danger signs for pneumonia | Hermida et al. (1999) | EX (pt) vs DO (exp) | 10 | - | - | 75 | 93 | - | - | - | - | - |
| Counselled mother on family planning | Hermida et al. (1999) | EX (pt) vs DO (exp) | 21 | - | - | 57 | 96 | - | - | - | - | - |
| General assessment |  |  |  |  |  |  |  |  |  |  |  |  |
| Asked about ability to drink | Franco et al. (2002) | EX (pt) vs DO (exp) | 9 | 72 | 0.189 | - | - | - | - | - | - | - |
| Asked about convulsions | Franco et al. (2002) | EX (pt) vs DO (exp) | 5 | 91 | 0.381 | - | - | - | - | - | - | - |
| Assessed consciousness | Franco et al. (2002) | EX (pt) vs DO (exp) | 34 | 43 | -0.284 | - | - | - | - | - | - | - |
| Asked about presence of cough | Franco et al. (2002) | EX (pt) vs DO (exp) | 68 | 83 | 0.634 | - | - | - | - | - | - | - |
| Asked about presence of diarrhoea | Franco et al. (2002) | EX (pt) vs DO (exp) | 51 | 77 | 0.547 | - | - | - | - | - | - | - |
| Asked about presence of fever | Franco et al. (2002) | EX (pt) vs DO (exp) | 68 | 80 | 0.526 | - | - | - | - | - | - | - |
| Checked immunization status | Franco et al. (2002) | EX (pt) vs DO (exp) | 94 | 87 | 0.134 | - | - | - | - | - | - | - |
| Assessed growth | Franco et al. (2002) | EX (pt) vs DO (exp) | 95 | 95 | 0.231 | - | - | - | - | - | - | - |
| Weighed child | Franco et al. (2002) | EX (pt) vs DO (exp) | 74 | 87 | 0.684 | - | - | - | - | - | - | - |
| Plotted weight | Franco et al. (2002) | EX (pt) vs DO (exp) | 97 | 97 | 0.453 | - | - | - | - | - | - | - |
| Case management of cough |  |  |  |  |  |  |  |  |  |  |  |  |
| Asked about duration of cough | Franco et al. (2002) | EX (pt) vs DO (exp) | 76 | 79 | 0.464 | - | - | - | - | - | - | - |
| Asked about difficult breathing | Franco et al. (2002) | EX (pt) vs DO (exp) | 33 | 72 | 0.347 | - | - | - | - | - | - | - |
| Counted respiration rate | Franco et al. (2002) | EX (pt) vs DO (exp) | 10 | 82 | 0.231 | - | - | - | - | - | - | - |
| Observed for chest indrawing | Franco et al. (2002) | EX (pt) vs DO (exp) | 47 | 77 | 0.550 | - | - | - | - | - | - | - |
| Told diagnosis respiratory infection | Franco et al. (2002) | EX (pt) vs DO (exp) | 78 | 48 | 0.114 | - | - | - | - | - | - | - |
| Gave appropriate treatment for cough | Franco et al. (2002) | EX (pt) vs DO (exp) | 57 | 88 | 0.755 | - | - | - | - | - | - | - |
| Told to feed during illness | Franco et al. (2002) | EX (pt) vs DO (exp) | 31 | 72 | 0.416 | - | - | - | - | - | - | - |
| Told to increase fluids | Franco et al. (2002) | EX (pt) vs DO (exp) | 34 | 70 | 0.369 | - | - | - | - | - | - | - |
| Advised to relieve cough | Franco et al. (2002) | EX (pt) vs DO (exp) | 8 | 88 | 0.238 | - | - | - | - | - | - | - |
| Told to return if breathing difficult | Franco et al. (2002) | EX (pt) vs DO (exp) | 16 | 83 | 0.496 | - | - | - | - | - | - | - |
| Case management of diarrhoea |  |  |  |  |  |  |  |  |  |  |  |  |
| Asked about duration of diarrhoea | Franco et al. (2002) | EX (pt) vs DO (exp) | 78 | 87 | 0.567 | - | - | - | - | - | - | - |
| Asked about blood in the stools | Franco et al. (2002) | EX (pt) vs DO (exp) | 49 | 74 | 0.477 | - | - | - | - | - | - | - |
| Pinched skin | Franco et al. (2002) | EX (pt) vs DO (exp) | 28 | 71 | 0.241 | - | - | - | - | - | - | - |
| Checked for sunken eyes | Franco et al. (2002) | EX (pt) vs DO (exp) | 58 | 61 | 0.228 | - | - | - | - | - | - | - |
| Checked fontanel (for children under 12 months of age) | Franco et al. (2002) | EX (pt) vs DO (exp) | 14 | 75 | 0.390 | - | - | - | - | - | - | - |
| Told diagnosis of diarrhoea | Franco et al. (2002) | EX (pt) vs DO (exp) | 74 | 49 | 0.133 | - | - | - | - | - | - | - |
| Gave appropriate treatment for diarrhoea | Franco et al. (2002) | EX (pt) vs DO (exp) | 79 | 97 | 0.906 | - | - | - | - | - | - | - |
| Told to feed during illness | Franco et al. (2002) | EX (pt) vs DO (exp) | 44 | 83 | 0.661 | - | - | - | - | - | - | - |
| Told to feed after illness | Franco et al. (2002) | EX (pt) vs DO (exp) | 37 | 72 | 0.415 | - | - | - | - | - | - | - |
| Told to increase fluids | Franco et al. (2002) | EX (pt) vs DO (exp) | 48 | 78 | 0.564 | - | - | - | - | - | - | - |
| Explained how to prepare oral rehydration solution | Franco et al. (2002) | EX (pt) vs DO (exp) | 62 | 69 | 0.286 | - | - | - | - | - | - | - |
| Told to return if dehydration signs | Franco et al. (2002) | EX (pt) vs DO (exp) | 18 | 78 | 0.393 | - | - | - | - | - | - | - |
| Case management of fever |  |  |  |  |  |  |  |  |  |  |  |  |
| Asked presence other symptoms | Franco et al. (2002) | EX (pt) vs DO (exp) | 70 | 68 | 0.111 | - | - | - | - | - | - | - |
| Asked about vomiting | Franco et al. (2002) | EX (pt) vs DO (exp) | 44 | 72 | 0.400 | - | - | - | - | - | - | - |
| Asked about any treatment already taken | Franco et al. (2002) | EX (pt) vs DO (exp) | 55 | 74 | 0.491 | - | - | - | - | - | - | - |
| Took temperature | Franco et al. (2002) | EX (pt) vs DO (exp) | 43 | 90 | 0.796 | - | - | - | - | - | - | - |
| Checked for anaemia | Franco et al. (2002) | EX (pt) vs DO (exp) | 63 | 70 | 0.384 | - | - | - | - | - | - | - |
| Told diagnosis of malaria | Franco et al. (2002) | EX (pt) vs DO (exp) | 81 | 53 | 0.180 | - | - | - | - | - | - | - |
| Gave appropriate treatment for malaria | Franco et al. (2002) | EX (pt) vs DO (exp) | 94 | 97 | 0.683 | - | - | - | - | - | - | - |
| Asked wait 30 minutes after taking treatment | Franco et al. (2002) | EX (pt) vs DO (exp) | 23 | 97 | 0.374 | - | - | - | - | - | - | - |
| Told to increase fluid intake | Franco et al. (2002) | EX (pt) vs DO (exp) | 36 | 71 | 0.380 | - | - | - | - | - | - | - |
| Advised sponge bath | Franco et al. (2002) | EX (pt) vs DO (exp) | 25 | 75 | 0.371 | - | - | - | - | - | - | - |
| Told return in 3 days if still ill | Franco et al. (2002) | EX (pt) vs DO (exp) | 26 | 73 | 0.309 | - | - | - | - | - | - | - |
| Diagnosis provided | Onishi et al. (2011)  (2005 data) | EX (pt) vs DO | 42 | - | - | 72 | 69 | - | - | 0.71 | - | - |
| Diagnosis provided | Onishi et al. (2011)  (2006 data) | EX (pt) vs DO | 45 | - | - | 72 | 63 | - | - | 0.68 | - | - |
| Diagnosis provided | Onishi et al. (2011)  (2007 data) | EX (pt) vs DO | 58 | - | - | 67 | 67 | - | - | 0.67 | - | - |
| Advised on what to do at home | Onishi et al. (2011)  (2005 data) | EX (pt) vs DO | 72 | - | - | 77 | 71 | - | - | 0.74 | - | - |
| Advised on what to do at home | Onishi et al. (2011)  (2006 data) | EX (pt) vs DO | 76 | - | - | 82 | 70 | - | - | 0.76 | - | - |
| Advised on what to do at home | Onishi et al. (2011)  (2007 data) | EX (pt) vs DO | 80 | - | - | 81 | 65 | - | - | 0.73 | - | - |
| Adverse reactions to medicine explained | Onishi et al. (2011)  (2005 data) | EX (pt) vs DO | 14 | - | - | 33 | 90 | - | - | 0.61 | - | - |
| Adverse reactions to medicine explained | Onishi et al. (2011)  (2006 data) | EX (pt) vs DO | 8 | - | - | 37 | 89 | - | - | 0.63 | - | - |
| Adverse reactions to medicine explained | Onishi et al. (2011)  (2007 data) | EX (pt) vs DO | 14 | - | - | 47 | 89 | - | - | 0.68 | - | - |
| Offered a time to return | Onishi et al. (2011)  (2005 data) | EX (pt) vs DO | 29 | - | - | 48 | 86 | - | - | 0.67 | - | - |
| Offered a time to return | Onishi et al. (2011)  (2006 data) | EX (pt) vs DO | 47 | - | - | 62 | 87 | - | - | 0.75 | - | - |
| Offered a time to return | Onishi et al. (2011)  (2007 data) | EX (pt) vs DO | 51 | - | - | 51 | 82 | - | - | 0.67 | - | - |
| Advised on signs to return to health facility | Onishi et al. (2011)  (2005 data) | EX (pt) vs DO | 31 | - | - | 77 | 72 | - | - | 0.74 | - | - |
| Advised on signs to return to health facility | Onishi et al. (2011)  (2006 data) | EX (pt) vs DO | 43 | - | - | 83 | 70 | - | - | 0.77 | - | - |
| Advised on signs to return to health facility | Onishi et al. (2011)  (2007 data) | EX (pt) vs DO | 47 | - | - | 83 | 65 | - | - | 0.74 | - | - |
| **Malaria case management** |  |  |  |  |  |  |  |  |  |  |  |  |
| Advice on dosage regimen | Pulford et al. (2014) | EX (pt) vs DO | 84 | - | - | 96 (94 to 98) | 54 (40 to 66) |  |  |  |  |  |
| Dietary advice | Pulford et al. (2014) | EX (pt) vs DO | 6 | - | - | 54 (42 to 66) | 94 (92 to 96) |  |  |  |  |  |
| Advice on adverse effects | Pulford et al. (2014) | EX (pt) vs DO | 3 | - | - | 36 (17 to 61) | 99 (98 to 99) |  |  |  |  |  |
| Advice on re-engagement | Pulford et al. (2014) | EX (pt) vs DO | 19 | - | - | 58 (49 to 66) | 75 (70 to 80) |  |  |  |  |  |
| Advice on malaria prevention | Pulford et al. (2014) | EX (pt) vs DO | 11 | - | - | 69 (61 to 77) | 97 (96 to 98) |  |  |  |  |  |
| Rapid diagnostic test/blood slide completed | Pulford et al. (2014) | EX (pt) vs DO | 29 | - | - | 94 (89 to 97) | 98 (96 to 99) |  |  |  |  |  |
| Prescription made | Pulford et al. (2014) | EX (pt) vs DO | 86 | - | - | 92 (88 to 94) | 98 (93 to 99) |  |  |  |  |  |
| Sulphadoxine-Pyrimethamine prescribed | Pulford et al. (2014) | EX (pt) vs DO | 72 | - | - | 84 (79 to 88) | 71 (63 to 83) |  |  |  |  |  |
| 1st dose ingested | Pulford et al. (2014) | EX (pt) vs DO | 51 | - | - | 96 (93 to 97) | 95 (92 to 97) |  |  |  |  |  |
| Take away dose | Pulford et al. (2014) | EX (pt) vs DO | 69 | - | - | 98 (97 to 99) | 88 (82 to 93) |  |  |  |  |  |
| **Antenatal care** |  |  |  |  |  |  |  |  |  |  |  |  |
| Counselling on danger signs of pregnancy complications* | Assaf (2018)  (Haiti) | EX (pt) vs DO | 51 (47 to 55) | 52 | 0.05 | 33 | 72 | 55 | 51 |  | 1.2 | 0.9 |
| Counselling on danger signs of pregnancy complications* | Assaf (2018)  (Malawi) | EX (pt) vs DO | 54 (49 to 59) | 60 | 0.20 | 61 | 60 | 64 | 56 |  | 1.5 | 0.7 |
| Counselling on danger signs of pregnancy complications* | Assaf (2018)  (Senegal) | EX (pt) vs DO | 38 (34 to 42) | 56 | 0.13 | 60 | 54 | 45 | 69 |  | 1.3 | 0.7 |
| **Postnatal care** |  |  |  |  |  |  |  |  |  |  |  |  |
| Blood pressure check | McCarthy et al. (2018)  (Kenya) | EX (pt) vs DO | 41.1 |  |  | 78.7 (70.6 to 85.5) | 83.9 (77.9 to 88.8) |  |  | 0.813 (0.769 to 0.857) |  |  |
| Blood pressure check | McCarthy et al. (2018)  (Swaziland) | EX (pt) vs DO | 74.6 |  |  | 83.5 (73.9 to 90.7) | 51.7 (32.5 to 70.6) |  |  | 0.676 (0.58 to 0.78) |  |  |
| Breast Exam | McCarthy et al. (2018)  (Kenya) | EX (pt) vs DO | 31.0 |  |  | 75.0 (64.9 to 83.4) | 87.1 (81.9 to 91.2) |  |  | 0.810 (0.761 to 0.860) |  |  |
| Breast Exam | McCarthy et al. (2018)  (Swaziland) | EX (pt) vs DO | 72.6 |  |  | 86.2 (77.1 to 92.7) | 66.7 (47.2 to 82.7) |  |  | 0.764 (0.670 to 0.860) |  |  |
| Examine abdomen | McCarthy et al. (2018)  (Kenya) | EX (pt) vs DO | 33.1 |  |  | 83.3 (73.2 to 90.8) | 83.1 (77.7 to 87.6) |  |  | 0.832 (0.784 to 0.880) |  |  |
| Examine abdomen | McCarthy et al. (2018)  (Swaziland) | EX (pt) vs DO | 64.3 |  |  | 75.0 (64.4 to 83.8) | 67.9 (47.6 to 84.1) |  |  | 0.714 (0.610 to 0.810) |  |  |
| Examine vagina | McCarthy et al. (2018)  (Kenya) | EX (pt) vs DO | 20.4 |  |  | 56.0 (34.9 to 75.6) | 82.7 (77.8 to 87.0) |  |  | 0.694 (0.592 to 0.795) |  |  |
| Examine vagina | McCarthy et al. (2018)  (Swaziland) | EX (pt) vs DO | 71.4 |  |  | 83.1 (73.3 to 90.5) | 62.1 (42.3 to 79.3) |  |  | 0.726 (0.630 to 0.820) |  |  |
| Screen for cervical cancer | McCarthy et al. (2018)  (Kenya) | EX (pt) vs DO | NA |  |  | NA | NA |  |  | NA |  |  |
| Screen for cervical cancer | McCarthy et al. (2018)  (Swaziland) | EX (pt) vs DO | 48.8 |  |  | 81.0 (58.1 to 94.6) | 58.0 (47.7 to 67.8) |  |  | 0.695 (0.600 to 0.790) |  |  |
| Check anaemia | McCarthy et al. (2018)  (Kenya) | EX (pt) vs DO | 29.0 |  |  | 60.0 (49.7 to 69.7) | 85.7 (80.2 to 90.1) |  |  | 0.729 (0.675 to 0.782) |  |  |
| Check anaemia | McCarthy et al. (2018)  (Swaziland) | EX (pt) vs DO | 38.9 |  |  | 42.5 (31.5 to 54.1) | 69.7 (51.3 to 84.4) |  |  | 0.561 (0.46 to 0.66) |  |  |
| Ask about excessive bleeding | McCarthy et al. (2018)  (Kenya) | EX (pt) vs DO | 40.8 |  |  | 83.8 (75.1 to 90.5) | 78.8 (72.8 to 84.0) |  |  | 0.813 (0.768 to 0.859) |  |  |
| Ask about excessive bleeding | McCarthy et al. (2018)  (Swaziland) | EX (pt) vs DO | 61.7 |  |  | 65.9 (49.4 to 79.9) | 40.5 (29.3 to 52.6) |  |  | 0.532 (0.440 to 0.620) |  |  |
| Discuss danger signs after birth | McCarthy et al. (2018)  (Kenya) | EX (pt) vs DO | 29.2 |  |  | 61.0 (51.6 to 69.9) | 89.5 (84.4 to 93.4) |  |  | 0.753 (0.704 to 0.802) |  |  |
| Discuss danger signs after birth | McCarthy et al. (2018)  (Swaziland) | EX (pt) vs DO | 49.6 |  |  | 58.3 (44.9 to 70.9) | 60.0 (45.9 to 73.0) |  |  | 0.592 (0.500 to 0.680) |  |  |
| Discuss STIs or HIV/AIDS | McCarthy et al. (2018)  (Kenya) | EX (pt) vs DO | 34.6 |  |  | 68.7 (59.4 to 77.0) | 84.5 (78.8 to 89.1) |  |  | 0.766 (0.717 to 0.815) |  |  |
| Discuss STIs or HIV/AIDS | McCarthy et al. (2018)  (Swaziland) | EX (pt) vs DO | 63.7 |  |  | 64.3 (50.4 to 76.6) | 36.8 (24.4 to 50.7) |  |  | 0.506 (0.420 to 0.600) |  |  |
| Discuss how soon after delivery a woman can get pregnant | McCarthy et al. (2018)  (Kenya) | EX (pt) vs DO | 25.8 |  |  | 48.5 (40.6 to 56.4) | 84.1 (80.0 to 87.7) |  |  | 0.663 (0.620 to 0.706) |  |  |
| Discuss how soon after delivery a woman can get pregnant | McCarthy et al. (2018)  (Swaziland) | EX (pt) vs DO | 25.9 |  |  | 32.6 (22.8 to 43.5) | 81.0 (70.9 to 88.7) |  |  | 0.568 (0.500 to 0.630) |  |  |
| Discuss return to fertility | McCarthy et al. (2018)  (Kenya) | EX (pt) vs DO | 22.8 |  |  | 48.5 (40.7 to 56.3) | 88.6 (84.9 to 91.6) |  |  | 0.685 (0.644 to 0.727) |  |  |
| Discuss return to fertility | McCarthy et al. (2018)  (Swaziland) | EX (pt) vs DO | 16.6 |  |  | 23.5 (15.0 to 34.0) | 90.5 (82.1 to 95.8) |  |  | 0.570 (0.510 to 0.630) |  |  |
| Discuss benefits of birth spacing | McCarthy et al. (2018)  (Kenya) | EX (pt) vs DO | 30.3 |  |  | 55.6 (47.1 to 64.0) | 89.9 (84.5 to 93.9) |  |  | 0.728 (0.681 to 0.774) |  |  |
| Discuss benefits of birth spacing | McCarthy et al. (2018)  (Swaziland) | EX (pt) vs DO | 35.3 |  |  | 42.1 (33.3 to 51.2) | 78.1 (66.0 to 87.5) |  |  | 0.601 (0.530 to 0.670) |  |  |
| Discuss return to sexual activity | McCarthy et al. (2018)  (Kenya) | EX (pt) vs DO | 15.9 |  |  | 50.0 (39.4 to 60.6) | 91.1 (88.0 to 93.5) |  |  | 0.705 (0.652 to 0.758) |  |  |
| Discuss return to sexual activity | McCarthy et al. (2018)  (Swaziland) | EX (pt) vs DO | 24.4 |  |  | 38.8 (28.4 to 50.0) | 89.7 (81.3 to 95.2) |  |  | 0.642 (0.580 to 0.700) |  |  |
| Discuss family planning method (including natural methods) | McCarthy et al. (2018)  (Kenya) | EX (pt) vs DO | 65.6 |  |  | 93.1 (88.8 to 96.1) | 77.0 (69.1 to 83.7) |  |  | 0.850 (0.811 to 0.889) |  |  |
| Discuss family planning method (including natural methods) | McCarthy et al. (2018)  (Swaziland) | EX (pt) vs DO | 78.8 |  |  | 78.0 (70.0 to 84.8) | 19.3 (10.0 to 31.9) |  |  | 0.487 (0.420 to 0.550) |  |  |
| Receive any modern family planning method | McCarthy et al. (2018)  (Kenya) | EX (pt) vs DO | NA |  |  | NA | NA |  |  | NA |  |  |
| Receive any modern family planning method | McCarthy et al. (2018)  (Swaziland) | EX (pt) vs DO | 65.0 |  |  | 79.5 (72.4 to 85.5) | 76.8 (63.6 to 87.0) |  |  | 0.781 (0.72 to 0.85) |  |  |
| Explain advantage/ disadvantage of chosen family planning method | McCarthy et al. (2018)  (Kenya) | EX (pt) vs DO | NA |  |  | NA | NA |  |  | NA |  |  |
| Explain advantage/ disadvantage of chosen family planning method | McCarthy et al. (2018)  (Swaziland) | EX (pt) vs DO | 41.1 |  |  | 53.7 (43.8 to 63.3) | 69.9 (61.0 to 77.9) |  |  | 0.618 (0.56 to 0.68) |  |  |
| Discuss breastfeeding/ feeding for baby | McCarthy et al. (2018)  (Kenya) | EX (pt) vs DO | 62.2 |  |  | 79.4 (74.8 to 83.5) | 72.6 (65.3 to 79.0) |  |  | 0.760 (0.720 to 0.799) |  |  |
| Discuss breastfeeding/ feeding for baby | McCarthy et al. (2018)  (Swaziland) | EX (pt) vs DO | 83.9 |  |  |  |  |  |  |  |  |  |
| Examine baby (undressed) | McCarthy et al. (2018)  (Kenya) | EX (pt) vs DO | 74.6 |  |  | 82.7 (76.5 to 87.9) | 37.7 (29.1 to 46.9) |  |  | 0.602 (0.551 to 0.653) |  |  |
| Examine baby (undressed) | McCarthy et al. (2018)  (Swaziland) | EX (pt) vs DO | 92.4 |  |  | 95.8 (89.6 to 98.8) | 21.7 (7.5 to 43.7) |  |  | 0.588 (0.500 to 0.680) |  |  |
| Weigh the baby | McCarthy et al. (2018)  (Kenya) | EX (pt) vs DO | 92.2 |  |  | 96.6 (93.6 to 98.4) | 33.3 (20.0 to 49.0) |  |  | 0.649 (0.579 to 0.720) |  |  |
| Weigh the baby | McCarthy et al. (2018)  (Swaziland) | EX (pt) vs DO | 96.6 |  |  |  |  |  |  |  |  |  |
| Immunise baby | McCarthy et al. (2018)  (Kenya) | EX (pt) vs DO | 87.7 |  |  | 96.2 (93.8 to 97.9) | 69.5 (56.1 to 80.8) |  |  | 0.829 (0.769 to 0.889) |  |  |
| Immunise baby | McCarthy et al. (2018)  (Swaziland) | EX (pt) vs DO | 87.1 |  |  | 93.2 (89.4 to 96.0) | 39.7 (27.0 to 53.4) |  |  | 0.664 (0.600 to 0.730) |  |  |
| Gave information on baby’s sickness signs | McCarthy et al. (2018)  (Kenya) | EX (pt) vs DO | 61.4 |  |  | 81.7 (74.9 to 87.3) | 56.0 (48.7 to 63.2) |  |  | 0.689 (0.643 to 0.735) |  |  |
| Gave information on baby’s sickness signs | McCarthy et al. (2018)  (Swaziland) | EX (pt) vs DO | 59.0 |  |  | 60.7 (51.0 to 69.8) | 44.4 (30.9 to 58.6) |  |  | 0.526 (0.440 to 0.610) |  |  |
| **Family planning service** |  |  |  |  |  |  |  |  |  |  |  |  |
| Discussed which method the client would prefer | Bessinger and Bertrand (2001) | Ex (pt) vs DO | 88 | 85 | 0.71 | - | - | - | - | - | - | - |
| Gave instruction on when to return | Bessinger and Bertrand (2001) | Ex (pt) vs DO | 89 | 83 | 0.66 | - | - | - | - | - | - | - |
| Asked the client if she has any concerns or problems | Bessinger and Bertrand (2001) | Ex (pt) vs DO | 86 | 79 | 0.57 | - | - | - | - | - | - | - |
| Discussed the client’s fertility intentions | Bessinger and Bertrand (2001) | Ex (pt) vs DO | 51 | 65 | 0.30 | - | - | - | - | - | - | - |
| Told the client how to use the contraceptive method | Bessinger and Bertrand (2001) | Ex (pt) vs DO | 87 | 85 | 0.69 | - | - | - | - | - | - | - |
| Mentioned sexually transmitted diseases or HIV/AIDS | Bessinger and Bertrand (2001) | Ex (pt) vs DO | 15 | 78 | 0.56 | - | - | - | - | - | - | - |
| Gave information on side effects | Bessinger and Bertrand (2001) | Ex (pt) vs DO | 74 | 74 | 0.48 | - | - | - | - | - | - | - |
| Encouraged dual method use (for new client receiving a non-barrier method) | Bessinger and Bertrand (2001) | Ex (pt) vs DO | 32 | 74 | 0.47 | - | - | - | - | - | - | - |
| Explained that method does not protect against HIV/AIDS (for new client receiving a non-barrier method) | Bessinger and Bertrand (2001) | Ex (pt) vs DO | 21 | 63 | 0.26 | - | - | - | - | - | - | - |
| Gave accurate information on the method accepted | Bessinger and Bertrand (2001) | Ex (pt) vs DO | 87 | 84 | 0.67 | - | - | - | - | - | - | - |
| Treated client with respect/courtesy | Bessinger and Bertrand (2001) | Ex (pt) vs DO | 99 | 99 | 0.98 | - | - | - | - | - | - | - |
| Client received method of choice | Bessinger and Bertrand (2001) | Ex (pt) vs DO | 83 | 90 | 0.80 | - | - | - | - | - | - | - |
| Saw the client in private for counselling | Bessinger and Bertrand (2001) | Ex (pt) vs DO | 95 | 88 | 0.75 | - | - | - | - | - | - | - |
| Offered privacy for pelvic examination/ intrauterine device insertion | Bessinger and Bertrand (2001) | Ex (pt) vs DO | 99 | 93 | 0.86 | - | - | - | - | - | - | - |
| **Family planning** |  |  |  |  |  |  |  |  |  |  |  |  |
| Discussed 2 or more methods with client | Tumlinson et al. (2014) | EX (pt) vs SP | 97 | 77 (63 to 93) | - | 80 (61 to 92) | NA | 96 (80 to 100) | 0 (0 to 46) |  | NA | NA |
| Asked client about her preferred method | Tumlinson et al. (2014) | EX (pt) vs SP | 97 | 93 (78 to 99) | - | 96 (82 to 100) | NA | 96 (82 to 100) | NA |  | NA | NA |
| Helped client select a method | Tumlinson et al. (2014) | EX (pt) vs SP | 61 | 45 (27 to 64) | - | 32 (13 to 57) | 67 (35 to 90) | 60 (26 to 88) | 38 (18 to 62) |  | 1.0 | 1.0 |
| Discussed side effects | Tumlinson et al. (2014) | EX (pt) vs SP | 61 | 61 (42 to 78) | - | 89 (67 to 99) | 17 (2 to 48) | 63 (42 to 81) | NA |  | 1.1 | 0.6 |
| Told client how to use selected method | Tumlinson et al. (2014) | EX (pt) vs SP | 68 | 68 (49 to 83) | - | 90 (70 to 99) | 20 (3 to 56) | 70 (50 to 86) | NA |  | 1.1 | 0.5 |
| Treat client with respect | Tumlinson et al. (2014) | EX (pt) vs SP | 87 | 87 (70 to 96) | - | 100 (87 to 100) | NA | 87 (70 to 96) | NA |  | NA | NA |
| Asked client about her reproductive goals | Tumlinson et al. (2014) | EX (pt) vs SP | 0 | 23 (10 to 41) | - | NA | 23 (10 to 41) | 0 (0 to 14) | 100 (59 to 100) |  | NA | NA |
| Asked client whether she has any questions | Tumlinson et al. (2014) | EX (pt) vs SP | 35 | 45 (27 to 64) | - | 91 (59 to 100) | 20 (8 to 44) | 38 (20 to 59) | NA |  | 1.1 | 0.5 |
| Told client when to return for resupply/ follow-up | Tumlinson et al. (2014) | EX (pt) vs SP | 80 | 73 (54 to 88) | - | 92 (73 to 99) | 0 (0 to 46) | 79 (59 to 92) | NA |  | 0.9 | NA |
| Told client what to do if she experiences problems | Tumlinson et al. (2014) | EX (pt) vs SP | 32 | 35 (19 to 55) | - | 80 (44 to 98) | 14 (3 to 36) | 31 (14 to 52) | NA |  | 0.9 | 1.4 |
| Offered client additional services | Tumlinson et al. (2014) | EX (pt) vs SP | 13 | 32 (17 to 51) | - | NA | 26 (11 to 46) | 13 (3 to 34) | 88 (47 to 100) |  | NA | NA |

^a^ Coefficient of correlation

^b^ Calculated based on data reported in the original paper

NA: not applicable (not evaluated) or not available (e.g. could not be calculated due to small numbers). -: not reported. SE: standard error

**Appendix Table 3. Performance of provider exit interview against direct observation or standardised patient**

|  |  |  |  | **Performance of the assessment method vs reference standard** | | | | | | | | |
| --- | --- | --- | --- | --- | --- | --- | --- | --- | --- | --- | --- | --- |
| **Quality item /indicator** | **Study** | **Comparison (assessment method vs reference standard)** | **Provider performance based on reference standard** | **Agreement (%)** | **Kappa** | **Sensitivity (%)** | **Specificity (%)** | **PPV (%)** | **NPV (%)** | **Area under ROC curve** | **LR+** | **LR-** |
| **Case management of sexually transmitted diseases** |  |  |  |  |  |  |  |  |  |  |  |  |
| Asked about onset of symptoms | Franco et al. (1997) | EX (pr) ^a^ vs DO ^c^ | 96 | 63 | -0.08 | - | - | - | - | - | - | - |
| Asked about onset of symptoms | Franco et al. (1997) | EX (pr) ^b^ vs DO ^c^ | 96 | 96 | 0.00 | - | - | - | - | - | - | - |
| Asked about contact with high-risk partners | Franco et al. (1997) | EX (pr) ^a^ vs DO ^c^ | 57 | 63 | 0.29 | - | - | - | - | - | - | - |
| Asked about contact with high-risk partners | Franco et al. (1997) | EX (pr) ^b^ vs DO ^c^ | 57 | 59 | 0.08 | - | - | - | - | - | - | - |
| Asked about recent new sexual partners | Franco et al. (1997) | EX (pr) ^a^ vs DO ^c^ | 67 | 55 | 0.14 | - | - | - | - | - | - | - |
| Asked about recent new sexual partners | Franco et al. (1997) | EX (pr) ^b^ vs DO ^c^ | 67 | 65 | 0.08 | - | - | - | - | - | - | - |
| Carried out any physical examination | Franco et al. (1997) | EX (pr) ^a^ vs DO ^c^ | 62 | 58 | -0.03 | - | - | - | - | - | - | - |
| Carried out any physical examination | Franco et al. (1997) | EX (pr) ^b^ vs DO ^c^ | 62 | 58 | -0.03 | - | - | - | - | - | - | - |
| Exposed patient fully | Franco et al. (1997) | EX (pr) ^a^ vs DO ^c^ | 100 | NA | NA | - | - | - | - | - | - | - |
| Exposed patient fully | Franco et al. (1997) | EX (pr) ^b^ vs DO ^c^ | 100 | NA | NA | - | - | - | - | - | - | - |
| Retracted foreskin (male patients) | Franco et al. (1997) | EX (pr) ^a^ vs DO ^c^ | 88 | 88 | 0.43 | - | - | - | - | - | - | - |
| Retracted foreskin (male patients) | Franco et al. (1997) | EX (pr) ^b^ vs DO ^c^ | 88 | NA | NA | - | - | - | - | - | - | - |
| Gave treatment for urethral discharge | Franco et al. (1997) | EX (pr) ^a^ vs DO ^c^ | 42 | 79 | 0.56 | - | - | - | - | - | - | - |
| Gave treatment for urethral discharge | Franco et al. (1997) | EX (pr) ^b^ vs DO ^c^ | 42 | NA | NA | - | - | - | - | - | - | - |
| Gave correct drugs/ dosages for urethral discharge | Franco et al. (1997) | EX (pr) ^a^ vs DO ^c^ | 32 | 82 | 0.56 | - | - | - | - | - | - | - |
| Gave correct drugs/ dosages for urethral discharge | Franco et al. (1997) | EX (pr) ^b^ vs DO ^c^ | 32 | NA | NA | - | - | - | - | - | - | - |
| Gave treatment for genital ulcer disease | Franco et al. (1997) | EX (pr) ^a^ vs DO ^c^ | 20 | 76 | 0.32 | - | - | - | - | - | - | - |
| Gave treatment for genital ulcer disease | Franco et al. (1997) | EX (pr) ^b^ vs DO ^c^ | 20 | NA | NA | - | - | - | - | - | - | - |
| Gave correct drugs/ dosages for genital ulcer disease | Franco et al. (1997) | EX (pr) ^a^ vs DO ^c^ | 20 | 76 | 0.27 | - | - | - | - | - | - | - |
| Gave correct drugs/ dosages for genital ulcer disease | Franco et al. (1997) | EX (pr) ^b^ vs DO ^c^ | 20 | NA | NA | - | - | - | - | - | - | - |
| Advised to finish treatment | Franco et al. (1997) | EX (pr) ^a^ vs DO ^c^ | 65 | 59 | 0.26 | - | - | - | - | - | - | - |
| Advised to finish treatment | Franco et al. (1997) | EX (pr) ^b^ vs DO ^c^ | 65 | NA | NA | - | - | - | - | - | - | - |
| Mentioned risk of AIDS | Franco et al. (1997) | EX (pr) ^a^ vs DO ^c^ | 57 | 61 | 0.20 | - | - | - | - | - | - | - |
| Mentioned risk of AIDS | Franco et al. (1997) | EX (pr) ^b^ vs DO ^c^ | 57 | 59 | 0.07 | - | - | - | - | - | - | - |
| Advised to use condoms | Franco et al. (1997) | EX (pr) ^a^ vs DO ^c^ | 51 | 57 | 0.13 | - | - | - | - | - | - | - |
| Advised to use condoms | Franco et al. (1997) | EX (pr) ^b^ vs DO ^c^ | 51 | 47 | -0.08 | - | - | - | - | - | - | - |
| Gave instructions on how to use condom | Franco et al. (1997) | EX (pr) ^a^ vs DO ^c^ | 4 | 92 | 0.30 | - | - | - | - | - | - | - |
| Gave instructions on how to use condom | Franco et al. (1997) | EX (pr) ^b^ vs DO ^c^ | 4 | 41 | 0.05 | - | - | - | - | - | - | - |
| Told to have partner treated | Franco et al. (1997) | EX (pr) ^a^ vs DO ^c^ | 86 | 57 | 0.08 | - | - | - | - | - | - | - |
| Told to have partner treated | Franco et al. (1997) | EX (pr) ^b^ vs DO ^c^ | 86 | NA | NA | - | - | - | - | - | - | - |
| Gave or prescribed condoms to patient | Franco et al. (1997) | EX (pr) ^a^ vs DO ^c^ | 19 | 31 | 0.03 | - | - | - | - | - | - | - |
| Gave or prescribed condoms to patient | Franco et al. (1997) | EX (pr) ^b^ vs DO ^c^ | 19 | NA | NA | - | - | - | - | - | - | - |
| **Acute childhood illnesses**** |  |  |  |  |  |  |  |  |  |  |  |  |
| Asked about difficult breathing | Franco et al. (2002) | EX (pr) vs DO | 53 | 67 | 0.318 | - | - | - | - | - | - | - |
| Asked about duration of diarrhoea | Franco et al. (2002) | EX (pr) vs DO | 90 | 79 | 0.298 | - | - | - | - | - | - | - |
| Pinched skin | Franco et al. (2002) | EX (pr) vs DO | 52 | 62 | 0.220 | - | - | - | - | - | - | - |
| Gave appropriate treatment for diarrhoea | Franco et al. (2002) | EX (pr) vs DO | 62 | 69 | 0.337 | - | - | - | - | - | - | - |
| Told to feed during illness | Franco et al. (2002) | EX (pr) vs DO | 50 | 71 | 0.429 | - | - | - | - | - | - | - |
| Told to feed after illness | Franco et al. (2002) | EX (pr) vs DO | 41 | 67 | 0.319 | - | - | - | - | - | - | - |
| Told to increase fluid intake | Franco et al. (2002) | EX (pr) vs DO | 64 | 82 | 0.602 | - | - | - | - | - | - | - |
| Explained how to prepare oral rehydration solution | Franco et al. (2002) | EX (pr) vs DO | 64 | 68 | 0.343 | - | - | - | - | - | - | - |
| Asked to wait 30 minutes after taking treatment | Franco et al. (2002) | EX (pr) vs DO | 47 | 67 | 0.312 | - | - | - | - | - | - | - |
| **Family planning** |  |  |  |  |  |  |  |  |  |  |  |  |
| Discussed 2 or more methods with client | Tumlinson et al. (2014) | EX (pr) vs SP | 96 | 94 (83 to 99) | - | 98 (89 to 100) | NA | 96 (86 to 100) | NA | - | NA | NA |
| Asked client about her preferred method | Tumlinson et al. (2014) | EX (pr) vs SP | 98 | 63 (48 to 77) | - | 63 (47 to 76) | NA | 100 (88 to 100) | 5 (0 to 26) |  | NA | NA |
| Helped client select a method | Tumlinson et al. (2014) | EX (pr) vs SP | 67 | 53 (38 to 68) | - | 76 (58 to 89) | 6 (0 to 30) | 63 (4 to 77) | 11 (0 to 48) |  | 0.8 | 4.0 |
| Discussed side effects | Tumlinson et al. (2014) | EX (pr) vs SP | 67 | 61 (46 to 75) | - | 82 (65 to 93) | 19 (4 to 46) | 68 (51 to 81) | 33 (8 to 70) |  | 1.0 | 0.9 |
| Discussed warning signs | Tumlinson et al. (2014) | EX (pr) vs SP | 6 | 80 (66 to 90) | - | NA | 83 (69 to 92) | 11 (0 to 48) | 95 (83 to 99) |  | NA | NA |
| Told client how to use selected method | Tumlinson et al. (2014) | EX (pr) vs SP | 73 | 49 (34 to 64) | - | 50 (33 to 67) | 46 (19 to 75) | 72 (51 to 88) | 25 (10 to 47) |  | 0.9 | 1.1 |
| Asked client about her reproductive goals | Tumlinson et al. (2014) | EX (pr) vs SP | 6 | 51 (36 to 66) | - | NA | 50 (35 to 65) | 8 (1 to 26) | 96 (79 to 100) |  | NA | NA |
| Told client when to return for resupply/ follow-up | Tumlinson et al. (2014) | EX (pr) vs SP | 78 | 45 (31 to 60) | - | 53 (36 to 69) | 18 (2 to 52) | 69 (49 to 58) | 10 (1 to 32) |  | 0.6 | 2.6 |

^a^ Based on spontaneous answers given by the provider in the exit interview

^b^ Based on spontaneous and probed answers given by the provider in the exit interview

^c^ For direct observation, a positive response was recorded if the provider was observed to have carried out the task on at least one patient

**The authors reported only ‘statistically significant agreement’ for the comparison between direct observation and provider interview

NA: not applicable (not evaluated) or not available (e.g. could not be calculated due to small numbers). -: not reported
